# Development of a Multilocus Sequence Typing Scheme for *Giardia intestinalis*

**DOI:** 10.3390/genes11070764

**Published:** 2020-07-08

**Authors:** Adriana Higuera, Marina Muñoz, Myriam Consuelo López, Patricia Reyes, Plutarco Urbano, Oswaldo Villalobos, Juan David Ramírez

**Affiliations:** 1 Grupo de Investigaciones Microbiológicas-UR (GIMUR), Departamento de Biología, Facultad de Ciencias Naturales, Universidad del Rosario, Bogotá 111211, Colombia; adriana.higuera@urosario.edu.co (A.H.); claudiamarina23@gmail.com (M.M.); 2 Departamento de Salud Pública, Universidad Nacional de Colombia, Bogotá 111321, Colombia; mlopezp@unal.edu.co (M.C.L.); preyes@unal.edu.co (P.R.); 3 Grupo de Investigaciones Biológicas de la Orinoquia, Unitrópico, Yopal 8500, Colombia; plurbanus@gmail.com; 4 Hospital Local Santa María de Mompox, Programas Especiales (Lepra y TB), Mompox, Bolívar 132560, Colombia; o.villalobosr@gmail.com

**Keywords:** *G. intestinalis*, genetic diversity, genetic structure, recombination

## Abstract

*Giardia intestinalis* is an intestinal protozoan most commonly found in humans. It has been grouped into 8 assemblages (A-H). Markers such as the glutamate dehydrogenase gene, triose phosphate isomerase and beta-giardin (β-giardin) have been widely used for genotyping. In addition, different genetic targets have been proposed as a valuable alternative to assess diversity and genetics of this microorganism. Thus, our objective was to evaluate new markers for the study of the diversity and intra-taxa genetic structure of *G. intestinalis* in silico and in DNA obtained from stool samples. We analysed nine constitutive genes in 80 complete genome sequences and in a group of 24 stool samples from Colombia. Allelic diversity was evaluated by locus and for the concatenated sequence of nine loci that could discriminate up to 53 alleles. Phylogenetic reconstructions allowed us to identify AI, AII and B assemblages. We found evidence of intra- and inter-assemblage recombination events. Population structure analysis showed genetic differentiation among the assemblages analysed.

## 1. Introduction

*Giardia intestinalis* (synonym *G. lamblia*, *G. duodenalis*), a single-celled eukaryotic protozoan, is the most common cause of parasitic diarrhoea in humans worldwide [1]. It infects approximately 2% of adults and between 6% and 8% of children in developed countries. About 33% of people have had giardiasis in developing countries [2]. Transmission of this protozoan is considered both zoonotic and zooanthroponotic since it is present in domestic [3] and wild animals [4]. The frequency of transmission among hosts is unclear [5]. Still, a risk for massive spread is known to exist. 

Different molecular tools [6,7,8] and genetic markers [9,10] with different mutation rates [11], have been used to evaluate the inter- and intra-specific variation of *G. intestinalis* [12,13], based mainly on 3 loci, β-giardin, triose phosphate isomerase and glutamate dehydrogenase [14,15]. These loci supported the identification of eight assemblages, termed A through H. These assemblages can be host-specific [16,17,18,19] and have allowed determination of assemblages A and B as most frequent [13], with assemblage B being most common in humans [20]. Assemblage B is also associated with more severe and prolonged disease and is considered the most virulent [3,13,21,22,23]. Sub-assemblages, such as AI, AII, AIII, BIII and BIV [24,25], have also been established using the above loci. However, despite their utility, these typing markers have produced contradictory results [14] or low resolution [24] when identifying assemblages in some samples. 

Some authors have proposed the study of new genes [26], which, when added to typically used markers, might better elucidate intra-specific diversity, along with nucleotide heterozygosity, allelic divergence and even recombination processes and inter/intra-genetic exchange [24,26,27,28,29]. This possibility is studied through single nucleotide polymorphisms and phylogenetic analyses and even comparative genomics. Such analyses indicate that sexual or meiotic processes may promote the generation of more virulent strains or expand their host range [26]. Additionally, exploring other genetic markers will allow characterisation of sub-assemblages not clearly established in assemblage E and perhaps others, and provide needed information on the substructures of assemblages C, D, F and G [21]. 

Evaluation of additional regions of the genome of *G. intestinalis* is needed to identify new markers for understanding its diversity and evolution. Such markers should possess sufficient discriminatory power to establish groupings related to epidemiological factors. Thus, investigation of new markers should focus on detection and typing, and allow additional inference on reproduction, evolution, zoonotic potential and population structure [30,31,32]. Some studies show that multilocus sequences are useful for identifying species, genera and populations, characterising isolates with conserved genes with low variation, and thus establishing allelic profiles in study populations [33]. Initially, this tool was widely used for bacteria [33,34], and subsequently has been implemented with diploid eukaryotes [35,36,37,38] and fungi [39,40,41]. This is because, despite the availability of complete genome analysis, the Multilocus sequence typing (MLST) approach is more accessible and economic, together with the selection of suitable markers, it is possible to generate high-resolution information for analysis of genetic diversity and evolution, without the bias of complete information on the genome that includes regions that are not informative or exposed to different selection pressures, which could be useful in other types of studies.

Few studies on *G. intestinalis* are available that sought to address additional genetic markers. Yet, generating multilocus analyses is essential for understanding the genetic characteristics of circulating strains in different geographical regions and monitoring their evolution and adaptation. Such analysis will encourage the design of strategies to decrease infection incidence [21]. In the present study, we evaluated different coding loci for constitutive enzymes involved in metabolic pathways, such as glycolysis and the Tricarboxylic acid cycle (TCA), focused on identifying genotypic characteristics of *G. intestinalis* tested in publicly available whole-genome sequences (WGS) and subsequently analyse these markers in DNA from stool samples from some regions of Colombia. Our proposed new markers are capable of elucidating diversity, population structure and possible recombination events between and within *G. intestinalis* assemblages.

## 2. Results

### 2.1. Analysis of New Loci Using WGS Data

#### 2.1.1. Genetic Diversity of Housekeeping Genes

Sequences of each assemblage, AI (WB), AII (DH), B (GS and GS_B) and E (P15) (Supplementary Materials, Table S1) for various genes were aligned, and conserved regions among assemblages used for primer design (Supplementary Materials, Table S2). Initially, eight genes were chosen, based on established criteria (see methods below). We used target genes and associated primers to evaluate *Pyrophosphate-fructose 6-phosphate 1-phosphotransferase alpha subunit (PFP-ALPHA1)*, *Fructose-bisphosphate aldolase (FBA), Phosphoglycerate kinase (PGK), enolase, Acetyl-CoA synthetase (ACS), NADP-dependent malic enzyme (NADP-ME), Serine palmitoyltransferase 2 (SPT), Glutamate dehydrogenase (GDH)* and *Triose phosphate isomerase (TPI)* genes in 80 available WGS from *G. intestinalis*. (Supplementary Materials, Table S3). We mapped short reads from sequences of interest and obtained consensus sequences for each gene using Short read sequence typing 2 (SRST2) [42]. The SRST2 output file did not report the *GPI* gene in any genome, and it was excluded from the study. Thus, nine genes were used in the investigation of diversity.

The initial analysis of molecular characteristics of conserved genes, showed interesting differences among loci and the concatenated sequence. Multiple alignment of concatenated sequences with the nine loci over a length of 11,978 bp identified 2,651 polymorphic sites. The highest number haplotypes and haplotypic diversity were h:34 and 0.842, respectively. *NADP-ME* and *SPT* genes displayed the highest values for both nucleotide diversity (Pi: 0.107 and 0.108. respectively), Theta (per site) from Eta (0.060 and 0.059. respectively) and numbers of segregating polymorphic sites (464 and 458, respectively). The *SPT* showed the fewest haplotypes (h: 11) and haplotypic diversity (Hd: 0.686). In contrast, genes such as *GDH*, commonly used to type *G. intestinalis*, showed a nucleotide diversity value, Pi of 0.058 and a Theta (per site) from Eta of 0.034, both being the lowest among analysed loci (Table 1).

Assemblage diversity indices were also calculated. The concatenated alignments AI and B assemblages showed low nucleotide diversity compared to the AII assemblage, for which relatively higher values were obtained for most loci. In contrast, Hd among these assemblages, was slightly lower for AI. Further, genes *TPI* and *PGK* showed a value of zero for AI and AII assemblages for both nucleotide diversity and haplotypic diversity (Supplementary Materials, Tables S4–S6). Notably, positive results were observed for the evolutionary divergence parameter, Tajima D [43], for all loci, most of them statistically significant (*p* < 0.05; *p* < 0.01). However, when analysing assemblages, results for all loci in AI and B assemblages were negative (Supplementary Materials, Tables S4 and S6).

Finally, we evaluated the utility of loci, including numbers of polymorphisms, typing efficiency (TE) and discriminatory power (DP), using MLSTest software (CONICET, Salta, Argentina) [44]. Numbers of possible alleles found among loci used and their combinations, showed, for example, that the combination of all nine loci could identify up to 53 alleles. With six loci, up to 51 different alleles were detected, and with a single locus, particularly *ACS*, a minimum of 11 different alleles (Supplementary Materials, Table S7). We also compared TE and DP among all loci, finding that the *GDH* locus displays the highest TE. The highest DP, 0.885, for all loci was somewhat above the DP for *ACS*, 0.815 (Table 2).

#### 2.1.2. Phylogenetic Analysis and Recombination Signals

**Figure 1:**
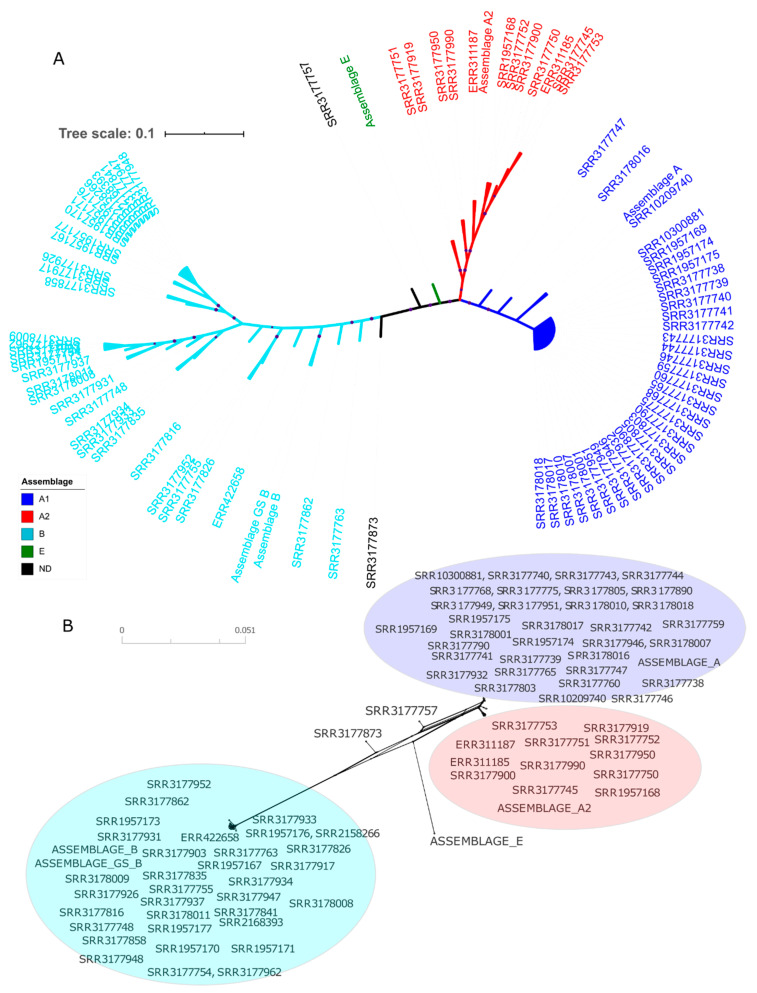
Phylogenetic reconstructions with sequences obtained from WGS data. Phylogenetic inferences were constructed from concatenated sequences of the nine selected genes. (**A**) Phylogenetic tree constructed from the alignment of concatenated sequences of all the genes evaluated. The tree was constructed with FastTree [45] software and visualized with ITOL [46] software. The maximum likelihood (ML) method was used under the Jukes Cantor nucleotide evolution model, with a Bootstrap of 1000 repetitions. A Bootstrap value greater than 90% is represented with a purple circle above each node. The colours indicate the assemblage to which the evaluated sequences belong (Blue: AI, Red: AII, Turquoise: B, Green: E and ND: not defined). (**B**) A phylogenetic network, using Splitstree software [47], was built with the Neighbornet algorithm; the colours correspond to assemblages. The access numbers of the genomes used are indicated.

The phylogenetic inferences constructed from sequences from concatenated genomic data and by gene, identified three main clusters, corresponding to *G. intestinalis* assemblages most commonly found in humans, the AI, AII and B (Figure 1A). The concatenated sequences of SRR3177757 and SRR3177873 genomes did not group within any established assemblage and are termed ND (not defined). Notably, comparing the position of these genomes in the phylogenetic tree, they coincide in grouping form, but the SRR3177757 genome shows evidence of inter-assemblage recombination, specifically among AI, AII and E. The SRR3177873 sequence, though showing reticulation signals, is located farther from these assemblages (Figure 1B). 

Phylogenies constructed with the *GDH* locus, in particular, demonstrate all three assemblages, along with evidence of intra-assemblage AII reticulation (Supplementary Materials, Figure S1). In contrast, trees generated with other loci, showed inconsistencies in tree topology due to locations of some evaluated genomes (Supplementary Materials, Figures S2–S9). For example, *FBA*, *NADP-ME* and *TPI* loci could not establish SRR3177751 and SRR3177919 genomes with certainty in an assemblage, and only a small recombination signal was observed in the *FBA* gene (Supplementary Materials, Figures S4, S7 and S9). Further, *enolase*, *PFP-ALPHA* and *PGK* loci did not clearly group all sequences with any assemblage but did group the sequences in the phylogenetic network (Supplementary Materials, Figures S3, S5 and S6).

Based on the comparison of clusters formed with concatenated sequences vs. clusters of each gene, *enolase* and *PFP-ALPHA1* genes presented the greatest number of inconsistencies in tree topology (Supplementary Materials, Figures S3 and S5). Still, the main clusters determined by maximum likelihood (ML) both for concatenated sequences (Figure 1A) and for each gene (Supplementary Materials, Figures S1A–S9A) were consistent with clusters found in phylogenetic networks using Neighbornet algorithm in Splitstree software [47] (Figure 1B and Supplementary Materials, Figures S1B–S9B). These findings support the presence of three established assemblages. 

In general, crosslinking signals were observed mainly between A and E assemblages and within the AII assemblage, indicating possible recombination events. We compared this evidence of recombination with calculated indices of minimum numbers of recombination events (Rm), for all evaluated alignments, using Dnasp software [48]. The highest Rm value was found with concatenated sequences, followed by *NADP-ME* and *ACS* genes (191, 42 and 30, respectively) (Table 1). This result is consistent with phylogenetic networks, except for the case of *NADP-ME* (Supplementary Materials, Figure S7). Further, we obtained 11 different recombinants in a search for recombination sites among concatenated sequences. The most frequent was in the SRR3177873 sequence, with breakpoints at different positions of the alignment, depending on parental sequences (Supplementary Materials, Figure S10).

**Figure 2:**
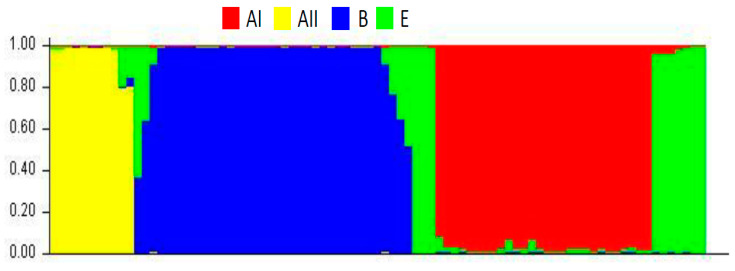
Analysis of STRUCTURE with the genome sequences. K = 4 populations were established a priori, corresponding to assemblages AI, AII, B and E. Colours indicate respective assemblages.

After phylogenetic topologies were generated and inter- and intra-assemblage crosslinks identified, a second analysis was developed in STRUCTURE [49,50]. K = 4 populations were established a priori. Clear signs of admixture between pre-established populations were observed (Figure 2). Populations are distinct, yet admixture is observed between assemblage E and the other assemblages, possibly by genetic interchange. Next, we used RDP4 software [51], to identify possible recombination events and identify their origin. We found 17 unique recombination events. Eight were detected by at least four different methods, providing further support for the presence of the event. Both detection by each method, and recombinant genomes and their possible parents from the concatenated sequences are presented (Table 3). Recombinant genomes, SRR3177757 and SRR3177873, were detected by at least five different methods. Recombination score through alignment (by position) for all detected events was calculated (Supplementary Materials, Figure S10). 

**Figure 3:**
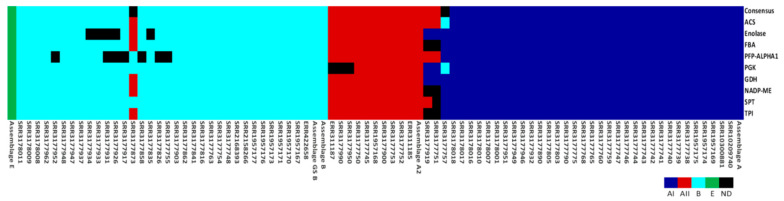
Allele plot constructed for with the alignment of the concatenated genomic sequences and for each gene. Colours indicate AI, AII, B, E, and ND (Not determined) assemblages. Each column corresponds to a genome and each row is a genetic marker. The assemblages were assigned depending on clusters obtained in the phylogenetic tree of each gene.

Finally, an allelic plot was constructed, using the classification of each genome in relation to the assemblage determined in the phylogenetic trees (bootstrap > 80), both by gene and concatenated sequence. (Figure 3). The presence of different colours in the same genome indicates inconsistencies in phylogenetic tree topology and subsequently in assemblage assignment. Such findings are consistent with possible recombination signals observed in different analyses. For example, SRR3177873 sequences are grouped in the AII sub-assemblage by *ACS*, *enolase*, *FBA*, *GDH*, *NADP-ME* and *TPI*, but in assemblage B with *PFP-ALPHA1*, *PGK* and *SPT*. No assemblage could be assigned with the concatenated sequence. Both SRR317799 and SRR3177751 showed inconsistencies between AII and AI assemblages. Some genes in the assemblage were indeterminate.

#### 2.1.3. Population Structure

Statistics used for genetic differentiation between populations are shown in Table 4. Established populations correspond to AI, AII and B assemblages. We evaluated concatenation and individual sequences by locus. We found higher values for the Gst statistic for all loci when comparing AI vs. AII and AI vs. B assemblages with respect to AII vs. B assemblages. For example, we saw noticeably lower value for concatenated sequences when evaluating AII vs. B assemblages (0.038); Gst for the other two comparisons were 0.344 (AI vs. AII) and 0.301 (AI vs. B). In contrast, Kxy, the average ratio of nucleotide differences between populations, and Dxy, the number of average nucleotide substitutions between populations, were greatly increased for both concatenated and all loci, and between the AI vs. B and AII assemblages vs. B. Fst indices were relatively high (Fst > 0.25) [52] for all cases, indicating a structure with elevated genetic differentiation between populations, in this case, the assemblages.

### 2.2. Analysis of New Loci Using *G. intestinalis* in Stool Samples

#### 2.2.1. Stool Samples

We randomly chose 24 samples positive for *G. intestinalis*, collected in different regions of Colombia, from Amazonas [53], Casanare, Bolívar, and Córdoba, as reported elsewhere [54].

#### 2.2.2. Amplification of New Loci in DNA Samples

We experimentally evaluated primers designed on loci analysed in silico. Initially, we tested with DNA extracted from *G. intestinalis* axenic culture. All primers adequately amplified corresponding regions with each locus. A single band of the expected size was obtained for each marker (Supplementary Materials, Table S1). We then tested primers for each locus with a small set (*n* = 24) of positive samples. Of the 24 samples, 95.8% (*n* = 23) amplified *GDH*; 83.3% (*n* = 20) amplified *TPI*; 66.7% (*n* = 16), *ACS*; 29.2% (*n* = 7) *SPT*, and 50.0% (*n* = 12), *enolase*. Many other samples amplified the target genes, and a band of the expected size was evident. However, other bands of different sizes were also observed. Also, concentrations of products obtained after the polymerase chain reaction (PCR) were low in some cases, showing bands so thin that it was impossible to obtain results from these sequences or no amplification occurred. Poor quality electropherograms were obtained for the *NADP-ME* gene, and sequences for this gene were ignored. Finally, five genes (*GDH*, *TPI*, *ACS*, *SPT* and *enolase*) were evaluated with DNA from the 24 stool samples. At least three markers were amplified in each sample, and all five loci were amplified in a few samples. (Supplementary Materials, Table S8). These results were not included in the MLST analysis.

#### 2.2.3. Genetic Diversity and Phylogenetic Reconstruction

Diversity indices for *ACS*, *SPT*, *enolase*, *GDH* and *TPI* loci were calculated with sequences obtained from stool samples, together with consensus sequences from the in silico analysis (Table 1). The *ACS* locus has a higher number of polymorphic sites (S = 278), compared to other loci, and the *TPI* locus the lowest number (S = 133), even though it displays the largest number of sites analysed. Also, nucleotide diversity, Pi, for the *ACS* gene was 0.210, followed by *SPT* and *TPI* loci with estimates of 0.114 and 0.109, respectively. Similarly, the *ACS* locus presented the highest value for Theta (per site) from Eta. Thus, locus that shows the greatest diversity is *ACS*. It is important to highlight that calculation of D Tajima as a neutrality test produced almost universally negative values. Not all values statistically significant, but still may indicate population expansion, at least for those loci, D Tajima for the *TPI* locus was positive, suggesting balancing selection. Another index calculated for this data set, corresponding to Rm, indicates a high value (Rm = 52) for the *TPI* locus compared to other target genes (Table 1).

**Figure 4:**
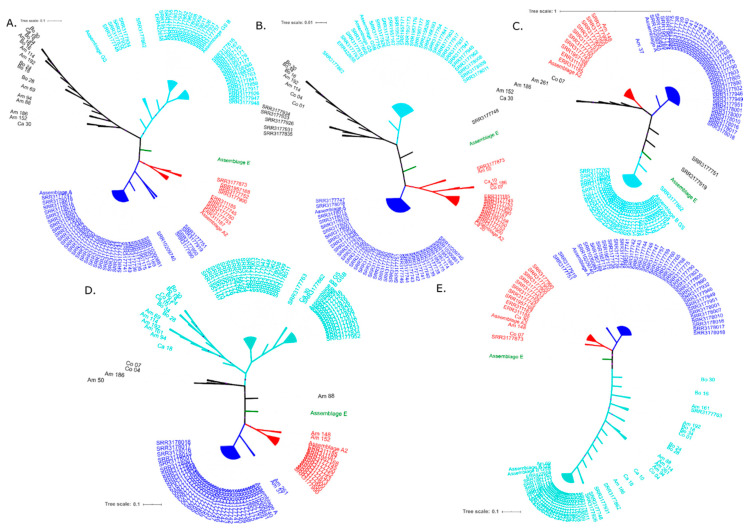
Gene trees including sequences obtained from human faeces as well as sequences extracted from public genomes. (**A**) *ACS*. (**B**) *Enolase*. (**C**) *SPT*. (**D**) *GDH*. (**E**) *TPI*. Phylogenetic inferences were constructed using maximum likelihood (ML) under the Jukes Cantor nucleotide evolution model, with 1000 bootstrap iterations. The tree was constructed with FastTree [45] software and visualized with ITOL [46] software. A Bootstrap value greater than 80% is represented with a purple circle above each node. The colours indicate the assemblage to which the evaluated sequences belong (Blue: AI. Red: AII. Turquoise: B. Green: E and ND: not defined). Access numbers of the genomes and origins of stool samples are indicated (AM: Amazonas. BO: Bolívar. CA: Casanare CO: Córdoba).

Subsequently, we prepared a phylogenetic reconstruction by gene that included the stool samples. *G. intestinalis* from stool samples form a cluster different from other sequences, except for the *TPI* gene. Three clusters corresponding to assemblages previously identified in silico analysis are observed (Figure 4). However, within the cluster corresponding to assemblage B, consensus sequences from the WGS data are closely grouped, and stool samples sequences are somewhat more distant. These observations suggest genetic differences that become more evident when other genes are included.

**Figure 5:**
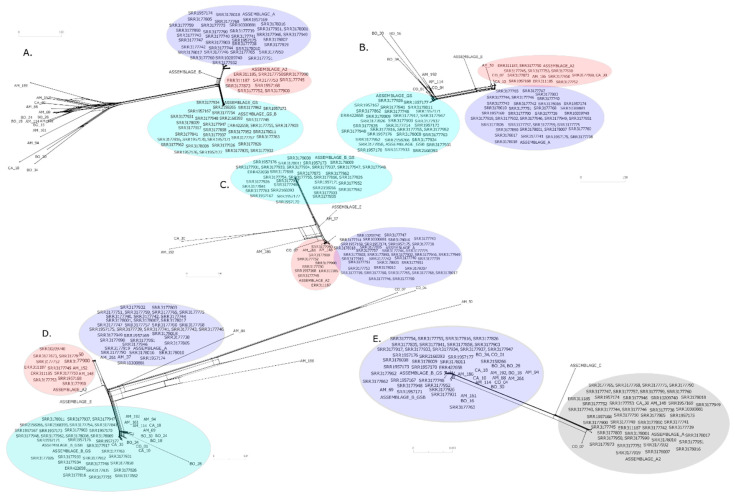
Phylogenetic network with SplitsTree [47] software, with sequences obtained from human faeces. (**A**) *ACS*. (**B**) *Enolase*. (**C**) *SPT*. (**D**) *GDH*. (**E**) *TPI*. Networks were built with the Neighbornet algorithm. Colours correspond to the assemblages (Blue: AI. Red: AII. Turquoise: B. Green: E. and ND: not defined). For the TPI gene, Assemblage A is highlighted in grey because AI and AII sub-assemblages are not discriminated. Access numbers of public genomes and origins of stool samples are indicated (AM: Amazonas. BO: Bolívar. CA: Casanare CO: Córdoba).

To explore possible explanations for the topology change in the obtained phylogenies, we constructed phylogenetic networks. Possible reticulation events are observed in the phylogenetic networks between DNA sequences derived from stool samples, both intra and inter-assemblage. These events are primarily associated with *ACS*, *GDH* and, to a lesser extent, *SPT* loci (Figure 5). *TPI* gene sequences could not discriminate between clusters of the AI and AII sub-assemblages, in contrast to other markers.

## 3. Discussion

The genes identified in the present study are useful for analysing calculated TE and DP. TE, an indicator of grouping of members with common characteristics, showed that proposed target genes are adequate to detect assemblages commonly found in human samples. DP, which allows differentiation of individuals belonging to different groups [44], showed that those genes are sufficient for identifying individuals that are slightly divergent from assemblages A and B. Considering these two parameters, implementing a typing scheme based on several loci is crucial. Markers must be adequate to assign an isolate to a "sequence type" or ST and powerful enough to differentiate one sequence type from another, but without discriminating to the point where each sequence becomes a different sequence type [55]. The latter could overestimate diversity and generate several STs. These results would make it difficult to establish phylogenetic relationships with epidemiological factors, as observed with *Candida albicans* [40]. Further, each ST corresponds to a relatively recent lineage, reflecting changes in the accessory genome, for example, a gene acquired parasexually [55]. Thus, we consider the inclusion of these new loci evaluated in this study useful for both typing and for studying the divergence within and between *Giardia* assemblages.

For *G. intestinalis,* no database built using a MLST scheme is available. Most studies, not surprisingly, use the same typing genes. The use of multiple loci to evaluate genetic characteristics of a microorganism has great advantages, such as ease of accessibility, basic bioinformatic requirements, and exponential enrichment of freely accessible databases [39]. Genomic data for *G. intestinalis* are available [56] and diversity studies have acquired information on regions of the genome that allow typing with sufficient DP [57]. In particular, we found that a combination of six different loci allows detection of up to 51 different alleles (Supplementary Materials, Table S7). Further, a combination of nine loci shows high DP followed by *ACS* and *PGK* markers (Table 2), though a greater number of polymorphisms were found with *ACS* and *NADP-ME*. However, no delimited genotypes or subgroups were observed among any established assemblages, consistent with previously reported MLGs using genes typically used for typing [58] and the previously reported MLST [24]. This background highlights the need for additional molecular targets to fully characterise the genetics of *G. intestinalis*. Genes in other regions of the genome will provide a comprehensive understanding of genetic diversity and genotypes.

Interestingly, in addition to diversity found in public sequences used for the MLST, haplotypic diversity was increased by including stool samples in the calculation of diversity indices. This result indicates that field samples are diverse in comparison to genomes evaluated in silico, as observed at the phylogenetic level. The finding likely reflects multiple sources of parasite transmission in the areas where samples were collected. Transmission may be affected by socioeconomic conditions in sampled populations [53], high dynamism of metropolitan areas, and high contact rates with different infected hosts, including symptomatic and asymptomatic patients, with different *G. intestinalis* assemblages. 

Evolutionary history was evaluated using neutrality analysis with the Tajima D test. We obtained positive D Tajima values for all loci analysing all genome sequences as a single population. From this approach, we can infer that the frequency of new alleles is low and that the population may be under balancing selection and contracting (Table 1). However, the assemblage analysis for all loci of the AI assemblage and the majority of loci of the assemblage B showed that the population is expanding (D Tajima < 0) (Supplementary Materials, Table S4; Table 1), which may be due to oral-faecal transmission that facilitates spread of cysts to new hosts [30]. Movement between hosts is crucial for gene flow and spread of rare alleles [59]. An expanding population was also inferred from results using stool samples. The use of constitutive loci allowed us to establish substantial diversity in each assemblage and population in general, despite the expectation that genes used are well conserved. These genes also allowed us to elucidate the evolutionary history of loci and the concatenation of all loci. Most values were significant, indicating that mutations may affect microorganism function and respond to selective pressure, as seen in studies using bacteria under an MLST scheme [60,61,62]. However, confirmation of this hypothesis will require increasing the number of individuals evaluated per assemblage to identify all evolutionary trends in *Giardia*.

Using phylogenetic analysis, we were able in most cases assign an assemblage for each consensus sequence in the in silico analysis, using loci independently and the concatenate of all loci. However, some sequences showed variations and did not clearly group with an assemblage. In some instances, clustering changed depending on the evaluated gene, showing confusion in delimiting criteria for assemblages (Figure 1 and Figure 3 and Supplementary Materials, Figures S1–S9). Our numbers of sequences for the in silico analysis was limited to 80, and the number of inconsistencies represents an important finding that must be considered when evaluating established assemblages. Sequences inclusion of stool samples increased the number of phylogenetic inconsistencies and diversity (Figure 4 and Table 1). Also, we found several subgroups within clusters, mainly for assemblage B, that likewise vary depending on the evaluated gene. Thus, substantial diversity exists within the assemblage. Such diversity could be related to a greater virulence [13] and evasion of host immunity [63] or may reflect the high heterozygosity found in this assemblage, how has been proposed in different studies of pathogenesis. An increase in the number of sequences for all assemblages, including non-A/B/E assemblages, would add certainty to intra-assemblage diversity. Our findings suggest reconsidering the classification of *G. intestinalis* exclusively in these assemblages, because of inconsistencies within clusters, high diversity, and alternative assemblage/cluster assignments depending on the analysed locus. 

When comparing ML trees results with phylogenetic networks, each assemblage shows important divergence with respect to AII. Such divergences in notable for A and B, and to a lesser extent, AI. Further, we observed reticulation signals and possible genetic exchange among assemblages, mainly between A and E, and for AI and AII (Figure 1B and Figure 5 and Supplementary Materials, Figures S1B–S9B). Inconsistencies in topologies of phylogenetic trees were previously reported [19]. These results suggest genetic exchanges, between isolates, and also with other microorganisms such as bacteria [56]. Recombination events evaluated with genomic data [29] are proposed, consistent with sequence analyses of several loci, mainly *GDH*, *TPI*, *β-giardin* and *small subunit ribosomal ribonucleic acid (ssurRNA)* [13]. Other genes with greater variability have also been proposed [24], based initially on changes in topologies of phylogenetic analyses [24,26,33,64]. Results obtained have opened debate on taxonomy and cell division processes in *G. intestinalis* [65], considering that, as a member of the diplomonads, it is typically asexual [66]. Also, changes in phylogenetic topology may be attributable to inadequate sampling, limited divergence, hybridisation, cryptic speciation with undocumented phenotypic differences, and incomplete lineage sorting (ILS) [67,68].

Further, admixture was observed among assemblages with the contribution of E assemblage alleles to AI, AII and B assemblages (Figure 2). Mixtures are mainly due to recombination events based on results with RDP (Table 3). Still, other genetic contributions should not be ruled out. The allelic plot shows, for some of sequences, no agreement between assigned assemblage and evaluated markers (Figure 3). No assemblage could be established for some genes with sufficient support (bootstrap > 80). These genes may belong in assemblages not evaluated in our study or may be the product of events that influence evolutionary dynamics of populations studied. Adaptive traits may be transferred that promote divergence due to events such as recombination, introgression or hybridisation [69]. Population structure statistics, such as the Fst were consistent with divergence among assemblages observed in phylogenetic networks (Figure 1B). Values higher than 0.8 were found for genetic structure among populations. We did not evaluate structure by geographic distances, and our results agree with studies based on haplotypic networks with the *TPI* gene from different continents. Genetic differentiation is reported between assemblage A and populations in Asia, Australia, and America. Moderate genetic differentiation is also seen with comparison using assemblages B and E. The latter case shows the dispersion of the same population of *G. intestinalis* [30]. We consider our results robust in support of the utility of proposed new loci to type *G. intestinalis*, and for investigating diversity, evolution, genetic structure and plausible genetic exchange events.

## 4. Materials and Methods 

### 4.1. Selection of New Genetic Markers and Design of Primers

Using sequenced, curated, and annotated genomes of *G. intestinalis* in the EuPathDB database: The Eukaryotic Pathogen Genomics Resource (GiardiaDB) (https://giardiadb.org/) (Supplementary Materials, Table S1), we searched for genes encoding constitutive enzymes that participate in metabolic processes, mainly glycolysis, alcoholic fermentation and TCA. Not all enzymes in the latter two are present in *G. intestinalis*. These proteins are highly conserved in eukaryotes, and most enzymes are reported for this microorganism [70]. Other enzymes in the sphingolipid biosynthesis pathway were also evaluated [71]. Specifically, we selected the following genetic targets from the glycolysis cycle: *Glucose-6-phosphate isomerase (GPI), Pyrophosphate-fructose 6-phosphate 1-phosphotransferase alpha subunit (PFP-ALPHA1), Fructose-bisphosphate aldolase (FBA), Phosphoglycerate kinase (PGK)* and *enolase*. We chose *Acetyl-CoA synthetase (ACS)* from the end of the alcoholic fermentation process, *NADP-dependent malic enzyme (NADP-ME)* from the TCA cycle and *Serine palmitoyltransferase 2 (SPT)* from lipid synthesis (Supplementary Materials, Table S1).

For bioinformatic design of primers, CDS sequences of orthologous and synthetic genes for these enzymes were downloaded for AI (isolate WB), AII (isolate DH), B (isolates GS and GS_B) and E (isolate P15) assemblages of eight genes (Supplementary Materials, Table S1). We aligned sequences of assemblages for each gene, using MUSCLE [72] implemented in MEGA 7.0. (Pennsylvania State University, PA, USA) [73]. We focused on identifying conserved regions between assemblages. Primers were designed using the Primer-BLAST tool (https://www.ncbi.nlm.nih.gov/tools/primer-blast/) and were chosen considering: (1) primers were located in conserved regions for all assemblages, (2) the final product had a size between 300 and 700 bp, (3) selected markers were single-copy genes in the *G. intestinalis* genome, and (4) information on dimers, formation of forks, melting temperature (Tm), per cent GC, size of initiator, size of the amplified region, and specificity were available. Single-copy genes were needed to estimate intra- and inter-assemblage allelic diversity, avoiding bias by recombination between copies of the genes. The fourth criterion was verified using Basic Local Alignment Search Tool (BLASTn).

Two approaches were used to verify single-copy genes. First, the Ortholog Groups of Protein Sequences (OrthoMCL DB) database, available at https://orthomcl.org/, was queried. The corresponding group of orthologs was searched for each protein to identify any alternate AA sequences. In the second, we used the CD-HIT comparative analysis algorithm, a tool for grouping and comparing biological sequences [74]. Sequences of each gene and each assemblage, previously downloaded from giardiadb.org in fasta format, were evaluated (Supplementary Materials, Table S1) and compared with transcript sequences available in NCBI. We looked numbers of sequences found for each gene with an identity ≥ 90% and Kmer = 2. Numbers of sequences found using these criteria within the same cluster were considered as possible variations in the number of gene copies.

### 4.2. In Silico Evaluation of Ten Genetic Markers 

A total of 10 loci were evaluated in 130 *G. intestinalis* genomes available online. Among these loci, we included genes commonly used for typing of *G. intestinalis*, glutamate dehydrogenase (GDH) and triose phosphate isomerase (TPI). Genes were used to evaluate genetic diversity, recombination, typing capacity, and discrimination of *G. intestinalis* in whole-genome sequencing (WGS) data available in the public database of The European Nucleotide Archive (ENA) (Supplementary Materials, Table S3).

We verified downloaded genomes using Kraken 2 software [75], which assesses DNA short-read sequences with a database of genomes from eukaryotes. One advantage of this approach is high sensitivity and speed, along with Kmers used for alignment to classify reads at different taxonomic levels [75]. We used hits greater than 80% as a cut-off point for data from short reads, obtaining 80 verified genomes for *G. intestinalis*. All other genomes were discarded (Supplementary Materials, Table S3).

Subsequently, we used the Short Read Sequencing Typing 2 (SRST2) tool [42] to extract genes of interest from the 80 selected WGS. This tool maps reads of each genome (sets of reads in fastq format) on a database of reference alleles in fasta format (the ten selected genes), to detect the presence of a gene or locus, and identify the allele that best matches the locus among all allelic sequences used for reference. The reference allele database was constructed using the sequences of each gene for each assemblage downloaded from EupathDB (UGA, Athens, GA, USA). Consensus sequences for each gene were obtained each public genome, together the STs were assigned, based on alleles that best matched each locus. The software did not yield consensus sequences for the GPI gene and it was eliminated from subsequent analyses. 

### 4.3. Utility of Selected Loci for Typing

Initially, consensus sequences of each gene and their concatenation were aligned using the multiple sequence alignment programme MAFFT v7 (Suita, Osaka, Japan) [76]. Subsequently, MLSTest software (CONICET, Salta, Argentina) was used to calculate numbers of alleles, typing efficiency (TE) and discriminatory power (DP) with 95% CI [44]. Alignments of consensus sequences from WGS data for each of nine markers were included as input data. An optimisation scheme was used to show optimal numbers of loci with different possible combinations and allelic profiles.

### 4.4. Phylogenetic Inferences and Recombination Signals

Phylogenetic trees were constructed from alignment of consensus sequences from WGS data (Supplementary Materials, Table S3). Subsequently, a phylogenetic tree was constructed by gene and with concatenation of all genes, using maximum likelihood under the Jukes Cantor nucleotide evolution model. The analysis used 1000 bootstrap replicates in FastTree 2.1 [45]. Each cluster was defined with bootstrap values ≥ 80.0%. Visualisation and editing used the online tool, Interactive Tree Of Life V4 (http://itol.embl.de) [46]. Additionally, phylogenetic networks were constructed to detect recombination signals between evaluated genes. The analysis used the SplitsTree5 programme [47], with the Neighbornet algorithm and 1000 iterations.

Once assemblages were established for each consensus sequence with each loci, an allelic plot schema was constructed with concatenated sequences and by gene. Each assemblage was represented in a different colour, the AI in blue, the AII in red, the B in turquoise, the E in green, and sequences that did not correspond to any assemblage were left in black. The scheme compares assemblages assigned to each consensus sequence with each gene, so that assemblage agreement among loci is represented. This scheme represents alleles found, such that the number of colours in the allelic plot represents the number of clusters discriminated by each marker, as reported in other studies [77].

To verify the existence of recombination events, we performed an additional analysis with the Recombination Detection Programme version 4 (RDP4) [51], using the alignment of concatenated sequences for the nine genes. RDP, GENECONV, BOOTSCAN, MaxChi Square (MaxChi), CHIMAERA, SISCAN and 3SEQ [78] were used, and recombination events described by multiple methods represent more robust results. To predict genetic admixture signals, we used the STRUCTURE 2.3.4 programme [49]. The number of established populations was K = 4, based on the four assemblages evaluated (AI, AII, B and E). We used 600,000 iterations of the Markov chain Monte Carlo algorithm with a Length of Burn-in Period of 60,000 iterations.

### 4.5. Indices of Diversity and Genetic Structure

All sequences in WGS data were used to calculate diversity indices, for each gene and concatenated sequence. Indices were also calculated with sequences grouped by assemblage. Input data in the DnaSP v.5 [48] programme (http://www.ub.edu/dnasp) were sequences aligned for each marker. Indices used included nucleotide diversity (Pi)—the average number of nucleotide differences per site between a pair of DNA sequences; Theta (per site) from the total number of mutations (Eta); numbers of polymorphic (segregating) sites (S); numbers of haplotypes (h); and haplotypic diversity (Hd). The latter index indicates the probability that two random haplotypes are different. Tajima D was calculated to determine if sequences evaluated reflected neutral variation or were involved in a selection process. This index indicates a balancing selection for positive values and a purifying selection for negative values [43]. The minimum number of recombination events (Rm) was also estimated. Some indices, such as haplotypic diversity and nucleotide diversity, are reported with their respective standard deviation.

Separate statistics for genetic differentiation among assemblages for each gene and concatenated sequence were also calculated. Assemblages that could not be defined for some sequences in some genes were assessed, as was the concatenated sequence from the E assemblage. Genetic differences were estimated using statistics based on haplotypes (Hs), nucleotide sequences (Ks), and several others that reflect gene flow from nucleotide sequences, including Wright’s F (Fst), Delta ST, Gamma ST, and Nst. Average number of nucleotide differences in pairs (Kxy), nucleotide substitutions per site (Dxy), net nucleotide substitutions per site (Da), and gene flow from haplotypes (Gst) were then calculated. DnaSP v.5 software [48] was used for the analysis (http://www.ub.edu/dnasp).

### 4.6. Assays from Human Stool Samples

#### 4.6.1. Ethical Statement

All subjects gave their informed consent for inclusion before they participated in the study. The study was conducted in accordance with the Declaration of Helsinki, and the protocol was approved by the Ethics Committee of the National University of Colombia (002-012-15 February 12, 2015) and the ethics committee of the Universidad del Rosario (registered in Act No. 394 of the CEI-UR). This project was conducted under the contract number RGE131 of access to genetic resources granted by the “Ministerio de Medio ambiente y Desarrollo sostenible”. 

#### 4.6.2. Study Population, Detection and Typing of *G. intestinalis*

Convenience sampling of human faeces samples was performed in the departments of Amazonas, Bolívar, Casanare, and Córdoba in Colombia. The collection, extraction and typing of samples positive for *G. intestinalis* was performed as described in Sánchez et al. [53] for samples from the Amazon and in Higuera et al. [54] for samples from Córdoba, Bolívar and Casanare. A small set of 24 DNAs from *G. intestinalis* positive samples were taken at random to evaluate markers assessed in silico. The number of samples by location were: 12 from Amazonas, six from Bolívar, three from Casanare and three from Córdoba. 

#### 4.6.3. DNA Marker Assay of Stool Samples

We amplified each locus from DNA extracted from *G. intestinalis* axenic cultures. PCR was performed in a final volume of 25 µL, containing 2 µL of quenched DNA, 12.5 µL of Go Taq Master Mix Green (Promega) (cat. No. M7122) (Madison, WIS, USA). at a final concentration of 1X and primers at a concentration of 1 µM each. Primers used for PCR are shown in Table 1, along with their respective expected band sizes. Thermal profile conditions for all loci were 95 °C for 5 min, 40 cycles of 95 °C for 1 min, 62 °C for 1 min, 72 °C for 1 min, and 10 min at 72 °C of final elongation. After verifying that all markers worked with control DNA, each *G. intestinalis* positive stool sample was amplified using the above conditions. All PCR products were verified by observation on 2% agarose gel, stained with SYBR Safe, Thermo Fisher Scientific (cat No. S33102) (MA, CA, USA). Each PCR product was purified with ExoSAP-IT®, Affymetrix ™ (cat. No. 15513687) (Göteborg, Sweden) following the manufacturer’s recommendations. Both chains of each product were sequenced with the Sanger method. Sequences were edited in MEGA 7.0 [73] to extract the fragment of interest. Once the sequence was cleaned, it was compared with publicly available sequences using the BLAST algorithm to verify that the fragment corresponded to the expected taxonomic unit.

#### 4.6.4. Phylogenetic Reconstructions and Diversity Indices from Stool Samples

Trees and phylogenetic networks were constructed with sequences obtained for each locus. Consensus sequences from the SRST2 tool output were concatenated with sequences obtained from human faeces of Colombian origin. This process used the same procedures described above for phylogenetic reconstructions. Diversity indices were also calculated by gene with the Dnasp v.5 programme [48].

## 5. Conclusions

We highlight loci useful of an MLST scheme for typing of *G. intestinalis*. These loci can also be used as alternatives to and supplements for genomic studies of *Giardia* diversity. We demonstrate intra-taxa diversity and show both genetic structures for established assemblages, and admixture among populations due to genetic exchange, apparently by recombination among individuals. Analysis of proposed loci should extend to future studies that include genomic sequences of additional assemblages to define their diversity and population structure. Further, future studies might focus on increasing the number of samples to evaluate markers on a large scale and extend sampling and analysis to other hosts and water sources that may be sources/reservoirs of infection. Such investigations will help elucidate transmission dynamics of the pathogen. Finally, future studies should assess a broader set of stool samples using nested PCR to examine the usefulness of genetic markers developed in the present study.

## Figures and Tables

**Table 1:** Diversity indices obtained for the evaluated loci.

	Marker	No. of Nucleotide Sites	No. of Sequences	Total Number of Sites	S	h	Hd (SD)	Pi (SD)	Theta	Tajima’s D test	Rm
WGS data consensus sequences	Concatenated	11978	85	11496	2651	34	0.842 (0.036)	0.086 (0.003)	0.0488	2.676 *	191
	*ACS*	2190	85	1857	394	19	0.81 (0.032)	0.078 (0.002)	0.0451	2.550 *	30
	*Enolase*	1338	85	1252	267	13	0.761 (0.036)	0.071 (0.003)	0.0455	1.983	16
	*FBA*	972	85	937	180	12	0.762 (0.03)	0.07 (0.002)	0.0423	2.242 *	11
	*PFP-ALHA1*	1650	85	1635	392	15	0.756 (0.034)	0.092 (0.003)	0.05	2.911 **	23
	*PGK*	1230	85	1204	272	16	0.799 (0.033)	0.091 (0.002)	0.047	3.169 **	22
	*GDH*	1386	85	1345	224	16	0.781 (0.036)	0.058 (0.002)	0.034	2.362 *	17
	*NADP-ME*	1689	85	1625	464	15	0.791 (0.034)	0.107 (0.004)	0.06	2.641 *	42
	*SPT*	1665	85	1641	458	11	0.686 (0.031)	0.108 (0.003)	0.059	2.896 **	24
	*TPI*	774	85	774	194	14	0.74 (0.034)	0.095 (0.003)	0.053	2.718 **	24
Sequences obtained from stool samples	*ACS*	562	101	297	278	26	0.831 (0.025)	0.21 (0.027)	0.326	–1.201	38
	*Enolase*	428	97	350	162	19	0.756 (0.029)	0.077 (0.005)	0.108	–0.978	26
	*GDH*	365	108	227	192	21	0.716 (0.039)	0.091 (0.013)	0.249	–2.118 **	26
	*SPT*	487	91	175	134	10	0.579 (0.031)	0.114 (0.013)	0.221	–1.639	15
	*TPI*	450	106	448	133	21	0.793 (0.026)	0.109 (0.002)	0.061	2.531 *	52

Total number of sites (excluding sites with gaps/missing data); S: Number of polymorphic (segregating) sites; h: Number of Haplotypes; Hd: Haplotype (gene) diversity; Pi: Nucleotide diversity; SD: Standard Deviation; Theta (per site) from Eta; Rm: Minimum number of recombination events. * Statistical significance: *p* < 0.05; ** Statistical significance: *p* < 0.01.

**Table 2:** Calculation of typing efficiency and discriminatory power of evaluated loci.

Name	*ACS*	*Enolase*	*FBA*	*PFP-ALPHA1*	*PGK*	*GDH*	*NADP-ME*	*SPT*	*TPI*	All Loci
**Number of Alleles**	21	15	14	15	18	20	15	11	14	53
**Number of Polymorphisms**	727	353	215	407	298	265	528	482	194	3469
**Typing Efficiency**	0.029	0.042	0.065	0.037	0.06	0.075	0.028	0.023	0.072	0.041
**DP (95% Confidence Interval)**	0.815 (0.748–0.881)	0.768 (0.693–0.843)	0.775 (0.715–0.835)	0.756 (0.688–0.824)	0.801 (0.732–0.869)	0.796 (0.72–0.873)	0.791 (0.722–0.861)	0.686 (0.623–0.749)	0.74 (0.671–0.81)	0.885 (0.816–0.955)

**Table 3:** Detection of recombination events.

Event No.	Found in	Recombinants	Major Parent	Minor Parent	Detection Methods						
					RDP	GENE CONV	BootScan	MaxiChi	Chimaera	SiScan	3Seq
1	1	SRR3177873	SRR317790	SRR3177948	+	+	+	+	+	+	+
2	1	SRR3177873	SRR3177900	ERR422658	+	+	+	+	+	+	+
3	2	Assemblage_A2	SRR317799	SRR3177750	-	+	-	+	-	+	+
4	1	ERR422658	SRR3177816	Unknown	-	+	-	+	+	+	+
5	1	SRR3177873	SRR3177900	ERR422658	-	+	-	-	-	-	-
6	1	SRR3177873	SRR317790	Unknown	-	+	+	-	-	-	-
7	1	SRR3177751	SRR317791	Unknown	-	-	-	+	-	-	-
8	4	SRR3177750	SRR317790	Unknown	+	+	+	+	+	+	+
9	2	SRR3177950	SRR317790	SRR3177919	-	+	-	+	+	-	-
10	1	SRR3177873	SRR3177950	ERR422658	-	+	-	-	-	-	-
11	1	SRR3177757	SRR102097	ERR422658	-	+	+	+	+	+	+
12	1	SRR3177757	SRR102097	SRR3177816	-	+	-	+	+	+	+
13	10	SRR3177948	SRR3178011	SRR3177926	-	-	-	+	+	-	+
14	1	SRR3177862	Assemblage_B	Unknown	-	-	-	+	+	-	+
15	1	SRR3177931	Unknown	SRR3178011	-	-	-	+	-	-	+
16	5	SRR3177952	SRR3178011	SRR3177926	-	-	-	+	-	-	+
17	1	SRR3177763	Unknown	SRR3177926	+	-	-	+	+	+	+

**Table 4:** Genetic differentiation among populations with concatenated sequences and each locus.

	Assemblages		Hs	Ks	Kxy	Gst	DeltaSt	GammaSt	Nst	Fst	Dxy	Da
**Concatenated**	AI	AII	0.353	1.616	13.844	0.344	0.004	0.760	0.809	0.808	0.012	0.009
	AI	B	0.536	7.910	193.217	0.301	0.078	0.923	0.962	0.961	0.162	0.156
	AII	B	0.909	12.207	194.079	0.038	0.061	0.951	0.951	0.948	0.163	0.155
***ACS***	AI	AII	0.300	3.952	36.542	0.421	0.006	0.774	0.822	0.822	0.018	0.015
	AI	B	0.435	3.732	298.743	0.396	0.075	0.976	0.989	0.988	0.151	0.149
	AII	B	0.757	8.637	301.443	0.115	0.060	0.935	0.971	0.967	0.152	0.147
***Enolase***	AI	AII	0.192	0.689	12.086	0.533	0.003	0.858	0.947	0.947	0.010	0.009
	AI	B	0.306	1.244	177.760	0.516	0.069	0.986	0.993	0.993	0.141	0.140
	AII	B	0.561	1.532	178.279	0.241	0.055	0.979	0.994	0.993	0.142	0.141
***FBA***	AI	AII	0.083	0.426	10.610	0.799	0.004	0.905	0.932	0.932	0.011	0.011
	AI	B	0.349	4.646	124.082	0.485	0.064	0.930	0.963	0.963	0.132	0.128
	AII	B	0.526	7.123	127.157	0.307	0.049	0.869	0.960	0.959	0.136	0.130
***PFP-ALPHAI***	AI	AII	0.213	0.736	16.048	0.526	0.004	0.896	0.919	0.918	0.010	0.009
	AI	B	0.176	1.496	295.903	0.695	0.089	0.990	0.995	0.994	0.181	0.180
	AII	B	0.424	3.025	293.796	0.333	0.077	0.977	0.991	0.990	0.180	0.178
***PGK***	AI	AII	0.138	0.795	9.667	0.675	0.002	0.780	0.950	0.950	0.008	0.008
	AI	B	0.467	2.942	216.423	0.366	0.088	0.974	0.988	0.987	0.178	0.176
	AII	B	0.651	3.995	217.676	0.233	0.052	0.942	0.990	0.989	0.179	0.177
***GDH***	AI	AII	0.251	1.342	18.435	0.443	0.004	0.829	0.876	0.875	0.014	0.012
	AI	B	0.402	1.580	152.200	0.423	0.056	0.980	0.990	0.989	0.113	0.112
	AII	B	0.724	3.149	150.617	0.127	0.042	0.949	0.979	0.977	0.112	0.109
***NADP-ME***	AI	AII	0.181	2.074	15.462	0.572	0.003	0.703	0.745	0.745	0.010	0.007
	AI	B	0.397	5.064	340.229	0.432	0.103	0.972	0.987	0.985	0.209	0.206
	AII	B	0.702	9.445	342.710	0.159	0.078	0.933	0.978	0.974	0.211	0.205
***SPT***	AI	AII	0.167	1.702	22.629	0.603	0.005	0.827	0.858	0.858	0.014	0.012
	AI	B	0.110	0.423	350.307	0.801	0.107	0.998	0.999	0.999	0.213	0.213
	AII	B	0.246	2.125	349.750	0.493	0.079	0.985	0.991	0.990	0.213	0.211
***TPI***	AI	AII	0.137	0.178	6.500	0.641	0.003	0.935	0.947	0.946	0.008	0.008
	AI	B	0.245	0.701	146.086	0.607	0.094	0.991	0.996	0.995	0.189	0.188
	AII	B	0.513	1.222	144.529	0.257	0.071	0.979	0.994	0.993	0.187	0.185

Hs: haplotype-based statistic; Ks: statistic based on nucleotide sequences; Kxy: average proportion of nucleotide differences between populations; Gst: genetic differentiation index based on the frequency of haplotypes; Dxy: average number of nucleotide substitutions per site between populations; Da: net nucleotide substitutions per site between populations.

## Data Availability

Sequences data that support the findings of this study were deposited in GenBank with accession codes: MN877659–MN877686, MN877687 and MN877710. The accession numbers for ACS, Enolase and SPT are MT499125–MT499159.

## Supplementary Materials

The following are available online at https://www.mdpi.com/2073-4425/11/7/764/s1. Figure S1: Phylogenetic reconstructions for the GDH locus from public data. (A). Phylogenetic tree built from concatenation of all genes evaluated. (B). Splits tree built with the Neighbornet algorithm; Figure S2: Phylogenetic reconstructions for the ACS locus from public data. (A). Phylogenetic tree built from concatenation of all genes evaluated. (B). Splits tree was built with the Neighbornet algorithm; Figure S3: Phylogenetic reconstructions for the enolase locus from public data. (A). Phylogenetic tree built from concatenation of all genes evaluated. (B). Splits tree built with the Neighbornet algorithm; Figure S4: Phylogenetic reconstructions for the FBA locus from public data. (A). Phylogenetic tree built from concatenation of all genes evaluated. (B). Splits tree built with the Neighbornet algorithm; Figure S5: Phylogenetic reconstructions for the PFP-ALPHA1 locus from public data. (A). Phylogenetic tree built from concatenation of all genes evaluated. (B). Splits tree built with the Neighbornet algorithm; Figure S6: Phylogenetic reconstructions for the PGK locus from public data. (A). Phylogenetic tree built from concatenation of all genes evaluated. (B). Splits tree built with the Neighbornet algorithm; Figure S7: Phylogenetic reconstructions for the NADP-ME locus from public data. (A). Phylogenetic tree built from concatenation of all genes evaluated. (B). Splits tree built with the Neighbornet algorithm; Figure S8: Phylogenetic reconstructions for the SPT locus from public data. (A). Phylogenetic tree built from concatenation of all genes evaluated. (B). Splits tree built with the Neighbornet algorithm; Figure S9: Phylogenetic reconstructions for the TPI locus from public data. (A). Phylogenetic tree built from concatenation of all genes evaluated. (B). Splits tree built with the Neighbornet algorithm; Figure S10: Graph of recombination score through alignment for all detected events. Events 1-17 are displayed. Table S1. Identification of GiardiaDB sequences and EC numbers of enzymes used as targets for the design of primers; Table S2. Sequences of primers designed for each gene evaluated; Table S3. Whole-genome sequencing information (WGS) data genomes available in the public database of The European Nucleotide Archive (ENA); Table S4. Diversity indices obtained for nine loci evaluated from the AI assemblage; Table S5. Diversity indices obtained for nine loci evaluated from the AII assemblage; Table S6. Diversity indices obtained for nine loci evaluated from assemblage B; Table S7. Analysis of scheme optimisation and the optimum number of loci; Table S8. Amplified loci for the 24 DNA samples evaluated.
