# Supplementary material for: Development of a Multilocus Sequence Typing Scheme for Giardia intestinalis
Source: Genes (Basel). 2020 Jul 8;11(7):764. doi: 10.3390/genes11070764 (PMC7397270; doi:10.3390/genes11070764)
Supplement: Supplementary file 1 [file genes-11-00764-s001.zip › Figure S2.pdf]

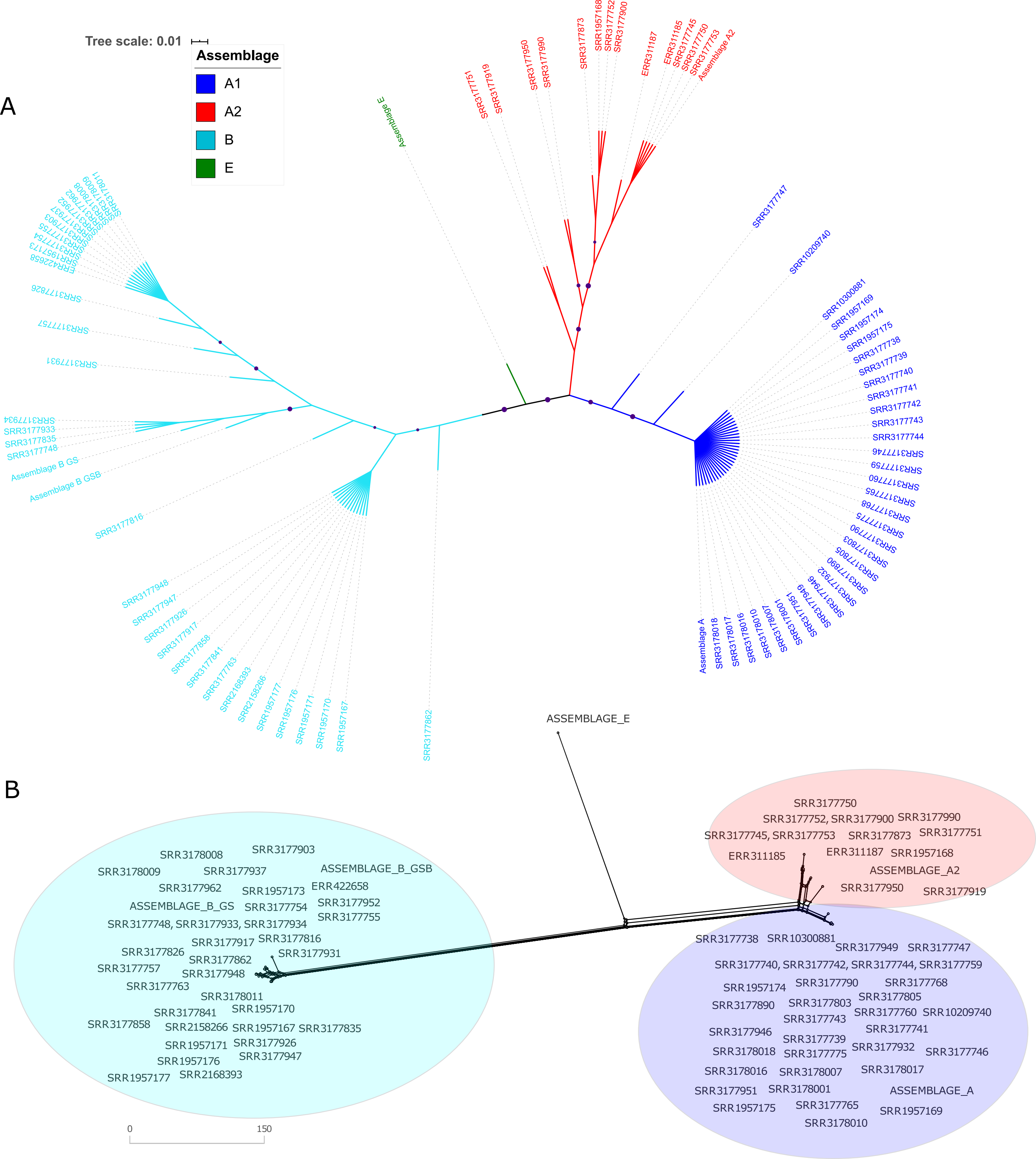

A
Tree scale: 0.01
Assemblage
A1
A2
B
E
Assemblage E
SRR3177751
SRR3177919
SRR3177950
SRR3177990
SRR3177873
SRR1957168
SRR3177752
SRR3177900
ERR311187
ERR311185
SRR3177745
SRR3177750
SRR3177753
Assemblage A2
SRR3177747
SRR10209740
SRR10300881
SRR1957169
SRR1957174
SRR1957175
SRR3177738
SRR3177739
SRR3177740
SRR3177741
SRR3177742
SRR3177743
SRR3177744
SRR3177746
SRR3177759
SRR3177760
SRR3177765
SRR3177768
SRR3177775
SRR3177790
SRR3177803
SRR3177805
SRR3177890
SRR3177932
SRR3177946
SRR3177949
SRR3177951
SRR3178001
SRR3178007
SRR3178010
SRR3178016
SRR3178017
SRR3178018
Assemblage A
SRR3177826
SRR3177757
SRR3177931
SRR3177934
SRR3177933
SRR3177835
SRR3177748
Assemblage B GS
Assemblage B GSB
SRR3177816
SRR3177948
SRR3177947
SRR3177926
SRR3177917
SRR3177858
SRR3177841
SRR3177763
SRR2168393
SRR2158266
SRR1957177
SRR1957176
SRR1957171
SRR1957170
SRR1957167
SRR3177862
ERR422658
B
ASSEMBLAGE_E
SRR3177750
SRR3177752, SRR3177900
SRR3177990
SRR3177745, SRR3177753
SRR3177873
SRR3177751
ERR311185
ERR311187
SRR1957168
ASSEMBLAGE_A2
SRR3177950
SRR3177919
SRR3177903
SRR3178008
SRR3178009
SRR3177937
ASSEMBLAGE_B_GSB
SRR3177962
SRR1957173
ERR422658
ASSEMBLAGE_B_GS
SRR3177754
SRR3177952
SRR3177755
SRR3177748, SRR3177933, SRR3177934
SRR3177917
SRR3177816
SRR3177826
SRR3177862
SRR3177931
SRR3177757
SRR3177948
SRR3177763
SRR3178011
SRR3177841
SRR1957170
SRR3177858
SRR2158266
SRR1957167
SRR3177835
SRR1957171
SRR3177926
SRR1957176
SRR3177947
SRR1957177
SRR2168393
0
150
SRR3177738
SRR10300881
SRR3177949
SRR3177747
SRR3177740, SRR3177742, SRR3177744, SRR3177759
SRR1957174
SRR3177790
SRR3177768
SRR3177890
SRR3177803
SRR3177805
SRR3177743
SRR3177760
SRR10209740
SRR3177946
SRR3177741
SRR3177739
SRR3178018
SRR3177775
SRR3177932
SRR3177746
SRR3178016
SRR3178007
SRR3178017
SRR3177951
SRR3178001
ASSEMBLAGE_A
SRR1957175
SRR3177765
SRR1957169
SRR3178010
