# Supplementary material for: Development of a Multilocus Sequence Typing Scheme for Giardia intestinalis
Source: Genes (Basel). 2020 Jul 8;11(7):764. doi: 10.3390/genes11070764 (PMC7397270; doi:10.3390/genes11070764)
Supplement: Supplementary file 1 [file genes-11-00764-s001.zip › Table S1.docx]

**Table S1****. Sequence identification from GiardiaDB and EC numbers of the enzymes used like target to primers design**

| Product description | Assemblage | Gene ID | Assemblage | Gene ID | Assemblage | Gene ID | Assemblage | Gene ID | Assemblage | Gene ID | Enzyme commission  numbers (KEGG) |
| --- | --- | --- | --- | --- | --- | --- | --- | --- | --- | --- | --- |
| ACS | A isolate WB | GL50803_13608 | A2 isolate DH | DHA2_13608 | B isolate GS_B | GSB_13608 | B isolate GS | GL50581_1568 | E isolate P15 | GLP15_4939 | EC 6.2.1.3 |
| Enolase | A isolate WB | GL50803_11118 | A2 isolate DH | DHA2_11118 | B isolate GS_B | GSB_11118 | B isolate GS | GL50581_4371 | E isolate P16 | GLP15_3306 | EC 4.2.1.11 |
| FBA | A isolate WB | GL50803_11043 | A2 isolate DH | DHA2_11043 | B isolate GS_B | GSB_11043 | B isolate GS | GL50581_4115 | E isolate P17 | GLP15_2077 | EC 4.1.2.13 |
| PFP-ALPHA1 | A isolate WB | GL50803_14993 | A2 isolate DH | DHA2_14993 | B isolate GS_B | GSB_14993 | B isolate GS | GL50581_1192 | E isolate P18 | GLP15_5089 | EC 2.7.1.90 |
| PGK | A isolate WB | GL50803_90872 | A2 isolate DH | DHA2_90872 | B isolate GS_B | GSB_90872 | B isolate GS | GL50581_4003 | E isolate P19 | GLP15_1654 | EC 2.7.2.3 |
| GDH | A isolate WB | GL50803_21942 | A2 isolate DH | DHA2_21942 | B isolate GS_B | GSB_21942 | B isolate GS | GL50581_4496 | E isolate P20 | GLP15_4574 | EC 1.4.1.4 |
| GPI | A isolate WB | GL50803_9115 | A2 isolate DH | DHA2_9115 | B isolate GS_B | GSB_9115 | B isolate GS | GL50581_1444 | E isolate P21 | GLP15_4116 | EC 5.3.1.9 |
| NADP-ME | A isolate WB | GL50803_14285 | A2 isolate DH | DHA2_14285 | B isolate GS_B | GSB_14285 | B isolate GS | GSB_14285 | E isolate P21 | GLP15_3168 | EC 1.1.1.40 |
| SPT | A isolate WB | GL50803_14374 | A2 isolate DH | DHA2_14374 | B isolate GS_B | GL50581_1960 | B isolate GS | GL50581_1960 | E isolate P22 | GLP15_1863 | EC 2.3.1.50 |
| TPI | A isolate WB | GL50803_93938 | A2 isolate DH | DHA2_93938 | B isolate GS_B | GSB_93938 | B isolate GS | GL50581_1369 | E isolate P23 | GLP15_4986 | EC 5.3.1.1 |
