# Supplementary material for: Development of a Multilocus Sequence Typing Scheme for Giardia intestinalis
Source: Genes (Basel). 2020 Jul 8;11(7):764. doi: 10.3390/genes11070764 (PMC7397270; doi:10.3390/genes11070764)
Supplement: Supplementary file 1 [file genes-11-00764-s001.zip › Table S2.docx]

| Table S2. Sequences of primers designed by each gen evaluated. | | |  |
| --- | --- | --- | --- |
| Loci | Sequence 5´-3´ | Size of band | Chromosome |
| Glucose-6-phosphate isomerase (GPI) | CAAATTCAGAAGGGGTGCTCGG | 557 | 5 |
|  | TTAGAGCACGCAATCCTACCTGCCAAAG |  |  |
| Pyrophosphate-fructose 6-phosphate 1-phosphotransferase alpha subunit (PFP-ALPHA1) | GTGGCCGCGACAAGATTGCTAAGCC | 578 | 5 |
|  | CGGTCCAGTGAGGCAGAAGTGTGT |  |  |
| Fructose-bisphosphate aldolase (FBA) | GTCCAGTTGAAGTCGCCCGTTA | 502 | 3 |
|  | GACCAGCGGGATGCCAGTAAGGTC |  |  |
| Phosphoglycerate kinase (PGK) | CCTCTTGGAGAATCTGCGCT | 415 | 2 |
|  | CAGTCTCTGCACCCTCTTTGTCGT |  |  |
| Enolase | CTGGAGCATCTACAGGGATGCACGA | 515 | 5 |
|  | GCAAGCCCAAACTCGTCCTTGAG |  |  |
| Acetyl-CoA synthetase (ACS) | TTACCCCGAACCTGAACTGC | 592 | 3 |
|  | GGAAGCGCCTCCTTTGTCTTGTC |  |  |
| NADP-dependent malic enzyme (NADP-ME) | ATAGGACAGCCAAAGCGTCAG | 582 | 5 |
|  | CATCTCGTCAGTGATCTTCTTTGC |  |  |
| Serine palmitoyltransferase 2 (SPT) | CCAGTGCCGTACAAAGCTAC | 593 | 3 |
|  | AGCACACGCAGCTCTTCAAAATCA |  |  |
