# Supplementary material for: Development of a Multilocus Sequence Typing Scheme for Giardia intestinalis
Source: Genes (Basel). 2020 Jul 8;11(7):764. doi: 10.3390/genes11070764 (PMC7397270; doi:10.3390/genes11070764)
Supplement: Supplementary file 1 [file genes-11-00764-s001.zip › Table S4.docx]

| Table S4. Diversity indices to 9 loci evaluated of AI assemblage | | | | | | | | | | |
| --- | --- | --- | --- | --- | --- | --- | --- | --- | --- | --- |
| Marker | **ACS** | **Enolase** | **FBA** | **PFP-ALPHA1** | **PGK** | **GDH** | **NADP-ME** | **SPT** | **TPI** | **Concatenated** |
| Number of nucleotide sites | 2190 | 1338 | 972 | 1650 | 1230 | 1386 | 1689 | 1665 | 774 | 11978 |
| Number of sequences | 34 | 38 | 35 | 35 | 36 | 37 | 35 | 35 | 35 | 34 |
| Total number of sites (excluding sites with gaps/missing data) | 2181 | 1289 | 938 | 1635 | 1230 | 1348 | 1674 | 1641 | 774 | 11894 |
| Number of polymorphic (segregating) sites, S | 9 | 12 | 2 | 1 | 10 | 6 | 2 | 2 | 0 | 16 |
| Number of Haplotypes, h | 3 | 3 | 2 | 2 | 4 | 3 | 2 | 2 | 1 | 3 |
| Haplotype (gene) diversity, Hd | 0,116 | 0.104 | 0,057 | 0,057 | 0,162 | 0,107 | 0,057 | 0,057 | 0 | 0,119 |
| Standard Deviation of Hd | 0,074 | 0.067 | 0,053 | 0,053 | 0,082 | 0,068 | 0,053 | 0,053 | 0 | 0,076 |
| Nucleotide diversity, Pi | 0,00024 | 0,00057 | 0,00012 | 0,00003 | 0,00079 | 0,00032 | 0,00007 | 0,00007 | 0 | 0,00008 |
| Standard deviation of Pi | 0,0002 | 0,00046 | 0,00011 | 0,00003 | 0,00047 | 0,0002 | 0,00006 | 0,00006 | 0 | 0,00007 |
| Theta (per site) from Eta | 0,00101 | 0,00222 | 0,00052 | 0,00015 | 0,00196 | 0,00107 | 0,00029 | 0,0003 | 0 | 0,00033 |
| Tajima´s D test | -2,29585** | -2,31403** | -1,4976 | -1,13552 | -1,81915* | -1,90973* | -1,49757 | -1,4976 | 0 | -2,5732*** |
| Minimum number of recombination events, Rm | 0 | 0 | 0 | 0 | 0 | 0 | 0 | 0 | 0 | 0 |
|  |  |  |  |  |  |  |  |  |  |  |

*Statistical significance: P < 0,05; ** Statistical significance: P < 0,01; *** Statistical significance: P < 0,001
