# Supplementary material for: Development of a Multilocus Sequence Typing Scheme for Giardia intestinalis
Source: Genes (Basel). 2020 Jul 8;11(7):764. doi: 10.3390/genes11070764 (PMC7397270; doi:10.3390/genes11070764)
Supplement: Supplementary file 1 [file genes-11-00764-s001.zip › Table S5.docx]

| Table S5. Diversity indices to 9 loci evaluated of AII assemblage | | | | | | | | | | |
| --- | --- | --- | --- | --- | --- | --- | --- | --- | --- | --- |
| Marker | **ACS** | **Enolase** | **FBA** | **PFP-ALPHA1** | **PGK** | **GDH** | **NADP-ME** | **SPT** | **TPI** | **Concatenated** |
| Number of nucleotide sites | 2190 | 1338 | 972 | 1650 | 1230 | 1386 | 1689 | 1665 | 774 | 11978 |
| Number of sequences | 14 | 11 | 12 | 13 | 8 | 12 | 12 | 12 | 12 | 13 |
| Total number of sites (excluding sites with gaps/missing data) | 2011 | 1338 | 972 | 1650 | 1230 | 1380 | 1625 | 1641 | 774 | 11879 |
| Number of polymorphic (segregating) sites, S | 43 | 1 | 9 | 12 | 0 | 12 | 20 | 24 | 2 | 167 |
| Number of Haplotypes, h | 6 | 2 | 2 | 4 | 1 | 5 | 3 | 3 | 3 | 10 |
| Haplotype (gene) diversity, Hd | 0,791 | 0,545 | 0,167 | 0,679 | 0 | 0,758 | 0,591 | 0,53 | 0,591 | 0,949 |
| Standard Deviation of Hd | 0,089 | 0,072 | 0,134 | 0,089 | 0 | 0,093 | 0,108 | 0,136 | 0,108 | 0,051 |
| Nucleotide diversity, Pi | 0,00644 | 0,0004 | 0,00154 | 0,00165 | 0 | 0,003 | 0,00479 | 0,00386 | 0,0009 | 0,00434 |
| Standard deviation of Pi | 0,00194 | 5E-05 | 0,00124 | 0,0007 | 0 | 0,0005 | 0,00096 | 0,00136 | 0,0002 | 0,00118 |
| Theta (per site) from Eta | 0,00672 | 0,0003 | 0,00307 | 0,00234 | 0 | 0,0029 | 0,00408 | 0,00484 | 0,0009 | 0,00453 |
| Tajima´s D test | -0,18240 | 1,44272 | -2,01608* | -1,21317 | 0 | 0,20403 | 0,77274 | -0,90266 | 0,15307 | -0,19610 |
| Minimum number of recombination events, Rm | 0 | 0 | 0 | 0 | 0 | 0 | 0 | 0 | 0 | 16 |

*Statistical significance: P < 0,05
