# Supplementary material for: Development of a Multilocus Sequence Typing Scheme for Giardia intestinalis
Source: Genes (Basel). 2020 Jul 8;11(7):764. doi: 10.3390/genes11070764 (PMC7397270; doi:10.3390/genes11070764)
Supplement: Supplementary file 1 [file genes-11-00764-s001.zip › Table S6.docx]

Table S6. Diversity indices to 9 loci evaluated of B assemblage

| Marker | ACS | Enolase | FBA | PFP-ALPHA1 | PGK | GDH | NADP-ME | SPT | TPI | Concatenated |
| --- | --- | --- | --- | --- | --- | --- | --- | --- | --- | --- |
| Number of nucleotide sites | 2190 | 1338 | 972 | 1650 | 1230 | 1386 | 1689 | 1665 | 774 | 11978 |
| Number of sequences | 35 | 30 | 36 | 29 | 37 | 35 | 35 | 36 | 35 | 35 |
| Total number of sites (excluding sites with gaps/missing data) | 2028 | 1307 | 971 | 1635 | 1215 | 1350 | 1689 | 1647 | 774 | 11877 |
| Number of polymorphic (segregating) sites, S | 15 | 10 | 145 | 20 | 29 | 11 | 44 | 14 | 19 | 180 |
| Number of Haplotypes, h | 9 | 5 | 6 | 6 | 9 | 7 | 7 | 4 | 8 | 18 |
| Haplotype (gene) diversity, Hd | 0,745 | 0,566 | 0,632 | 0,32 | 0,763 | 0,714 | 0,736 | 0,162 | 0,489 | 0,894 |
| Standard Deviation of Hd | 0,051 | 0,089 | 0,054 | 0,112 | 0,055 | 0,057 | 0,05 | 0,082 | 0,102 | 0,039 |
| Nucleotide diversity, Pi | 0,0026 | 0,00185 | 0,01011 | 0,00198 | 0,004 | 0,0021 | 0,00596 | 0,00047 | 0,00181 | 0,00299 |
| Standard deviation of Pi | 0,0002 | 0,00028 | 0,00746 | 0,00068 | 6,9E-05 | 0,0002 | 0,00075 | 0,00029 | 0,00084 | 0,00029 |
| Theta (per site) from Eta | 0,0018 | 0,00193 | 0,03626 | 0,00311 | 0,00572 | 0,002 | 0,00633 | 0,00205 | 0,00596 | 0,00368 |
| Tajima´s D test | 1,44860 | -0,12987 | -2,70298** | -1,28181 | -1,04469 | 0,15040 | -0,20736 | -2,47701** | -2,34350** | -0,71226 |
| Minimum number of recombination events, Rm | 2 | 0 | 5 | 1 | 2 | 2 | 8 | 0 | 0 | 25 |

** Statistical significance: P < 0,01
