# Supplementary material for: Development of a Multilocus Sequence Typing Scheme for Giardia intestinalis
Source: Genes (Basel). 2020 Jul 8;11(7):764. doi: 10.3390/genes11070764 (PMC7397270; doi:10.3390/genes11070764)
Supplement: Supplementary file 1 [file genes-11-00764-s001.zip › Table S7.docx]

| Table S7. Analysis of scheme optimization and the optimum number of loci | | | | |
| --- | --- | --- | --- | --- |
| Number of Loci (Number of Combinations) | Loci | Minimun Number  of alleles found | Mean Number  of alleles found | Maximun Number of alleles found |
| 1 (9) | ACS | 11 | 15,88889 | 21 |
| 2 (36) | PGK, GDH | 17 | 24,66667 | 32 |
| 3 (84) | PGK, GDH, NADP | 22 | 31,33333 | 40 |
| 4 (126) | ACS, PGK, GDH, NADP | 27 | 36,76191 | 46 |
| 5 (126) | ACS, Enolase, PGK, GDH, NADP ACS, PFP-ALPHA1, PGK, GDH, NADP | 32 | 41,25397 | 49 |
| 6 (84) | ACS, Enolase, PFP-ALPHA1, PGK, GDH, NADP ACS, PFP-ALPHA1, PGK, GDH, NADP, TPI | 37 | 45 | 51 |
| 7 (36) | ACS, Enolase, FBA, PFP-ALPHA1, PGK, GDH, NADP ACS, Enolase, PFP-ALPHA1, PGK, GDH, NADP, TPI ACS, FBA, PFP-ALPHA1, PGK, GDH, NADP, TPI | 43 | 48,13889 | 52 |
| 8 (9) | ACS, Enolase, FBA, PFP-ALPHA1, PGK, GDH, NADP, TPI | 48 | 50,77778 | 53 |
| 9 (1) | ACS, Enolase, FBA, PFP-ALPHA1, PGK, GDH, NADP, SPT, TPI | 53 | 53 | 53 |
