# Supplementary material for: Development of a Multilocus Sequence Typing Scheme for Giardia intestinalis
Source: Genes (Basel). 2020 Jul 8;11(7):764. doi: 10.3390/genes11070764 (PMC7397270; doi:10.3390/genes11070764)
Supplement: Supplementary file 1 [file genes-11-00764-s001.zip › Table S8.docx]

Table S8. Loci amplified for 24 DNA samples evaluated.

|  | Loci | | | | |
| --- | --- | --- | --- | --- | --- |
| Sample | ACS | Enolase | SPT | GDH | TPI |
| Am_114 | 339 | 413 |  | 57 | 20 |
| Am_148 |  |  | 403 | 71 | 30 |
| Am_152 | 340 |  | 404 | 74 |  |
| Am_161 | 341 |  |  | 80 | 34 |
| Am_186 | 342 | 414 | 405 | 89 | 39 |
| Am_192 | 343 | 415 |  | 94 | 41 |
| Am_261 |  |  | 409 | 117 | 51 |
| Am_37 |  |  | 401 | 24 |  |
| Am_50 |  | 412 |  | 29 |  |
| Am_69 | 336 |  |  | 35 | 11 |
| Am_88 | 337 |  |  | 44 | 14 |
| Am_94 | 338 |  |  | 47 | 16 |
| Bo_16 | 344 | 417 |  | 14 | 43 |
| Bo_18 | 345 |  |  | 15 | 44 |
| Bo_24 | 348 |  |  | 19 | 47 |
| Bo_28 | 349 |  |  | 22 | 49 |
| Bo_30 | 350 | 418 |  | 23 | 50 |
| Bo_34 | 351 | 419 |  | 25 | 52 |
| Ca_10 |  | 420 |  | 28 | 54 |
| Ca_18 | 354 |  |  | 30 | 59 |
| Ca_30 | 355 | 421 | 410 |  | 60 |
| Co_01 |  | 423 |  | 38 | 67 |
| Co_04 |  | 424 |  | 39 | 68 |
| Co_07 |  | 425 | 411 | 40 | 69 |

Los nombres de las muestras indican el departamento de origen, así: AM - Amazonas, BO - Bolívar, CA - Casanare, CO – Córdoba. Los cuadros rellenos corresponden a las muestras amplificadas.
