# Supplementary figures and images for: Development of a Multilocus Sequence Typing Scheme for Giardia intestinalis
Source: Genes (Basel). 2020 Jul 8;11(7):764. doi: 10.3390/genes11070764 (PMC7397270; doi:10.3390/genes11070764)

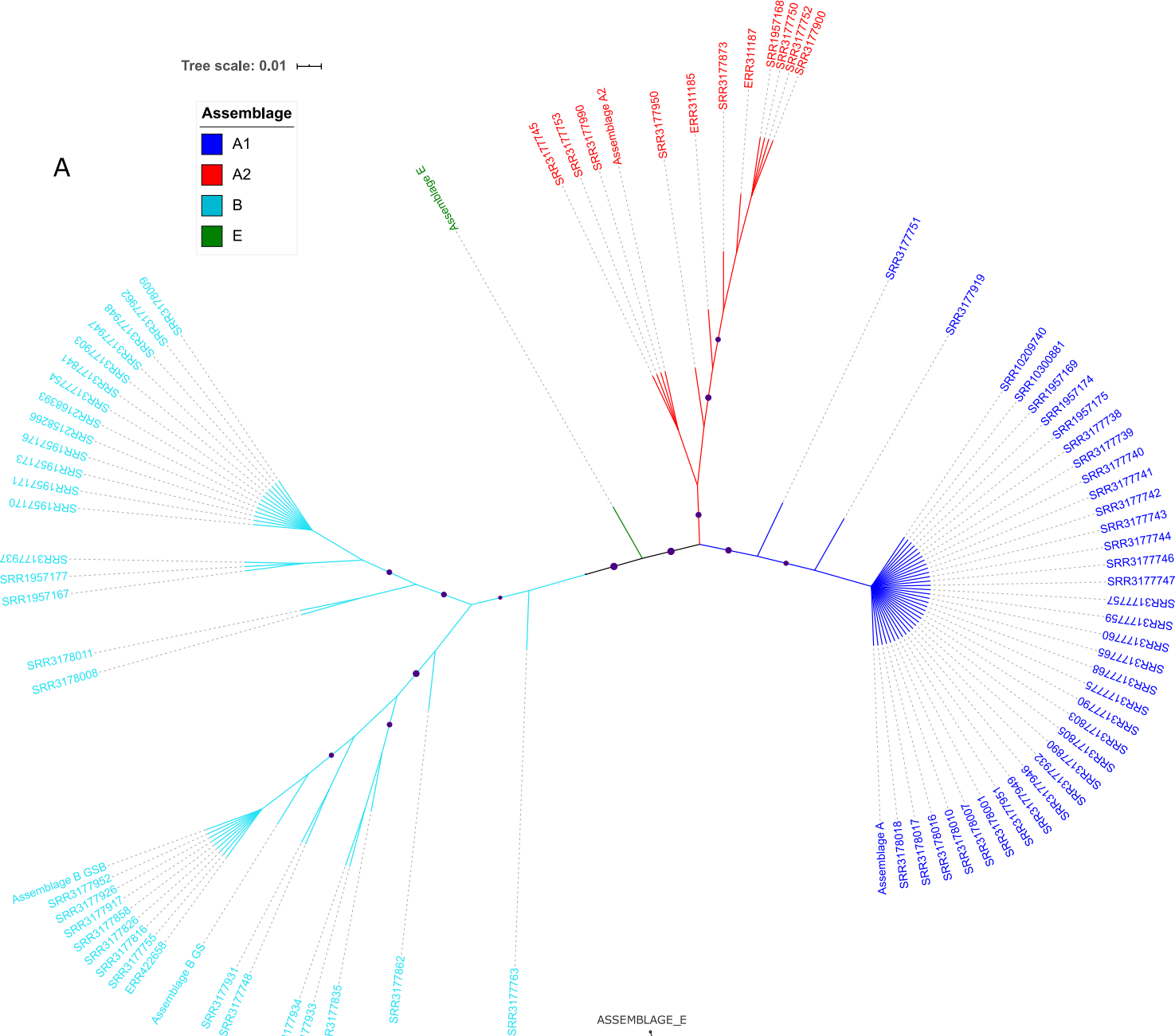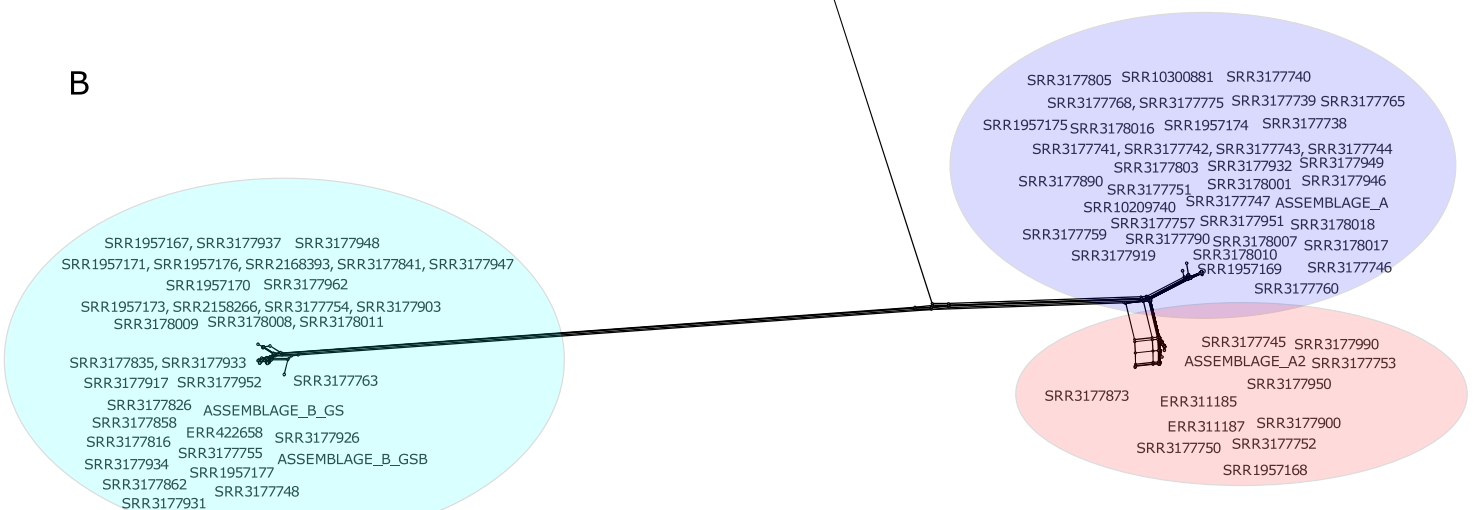

0 147.044

Supplement: Supplementary file 1 [file genes-11-00764-s001.zip › Figure S1.pdf]

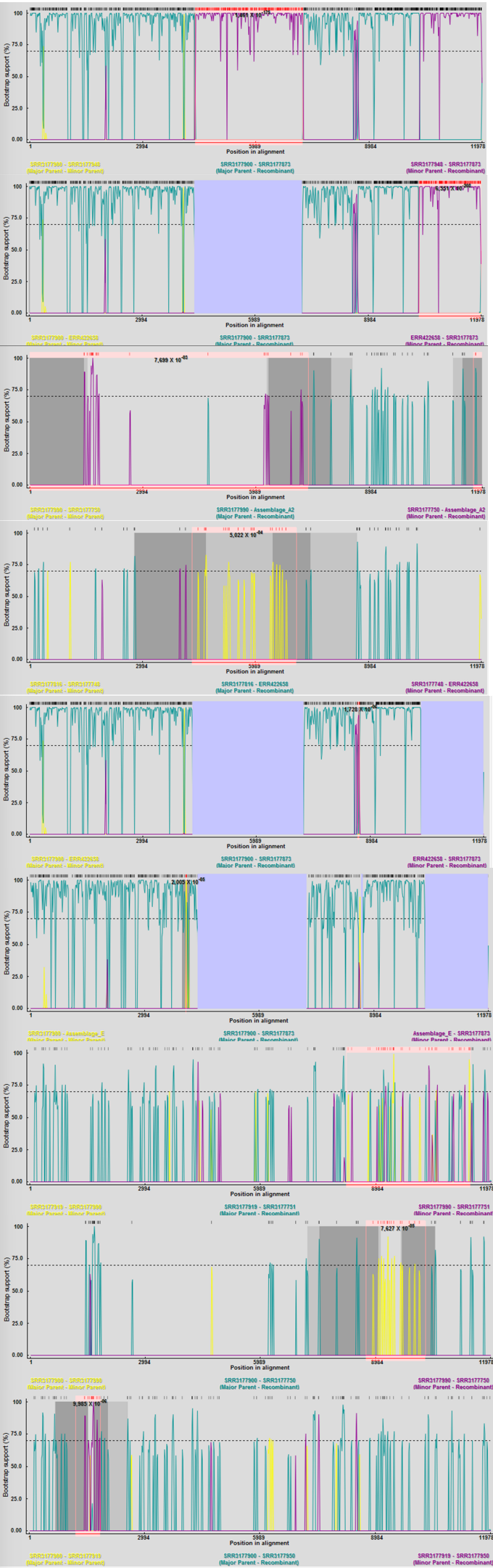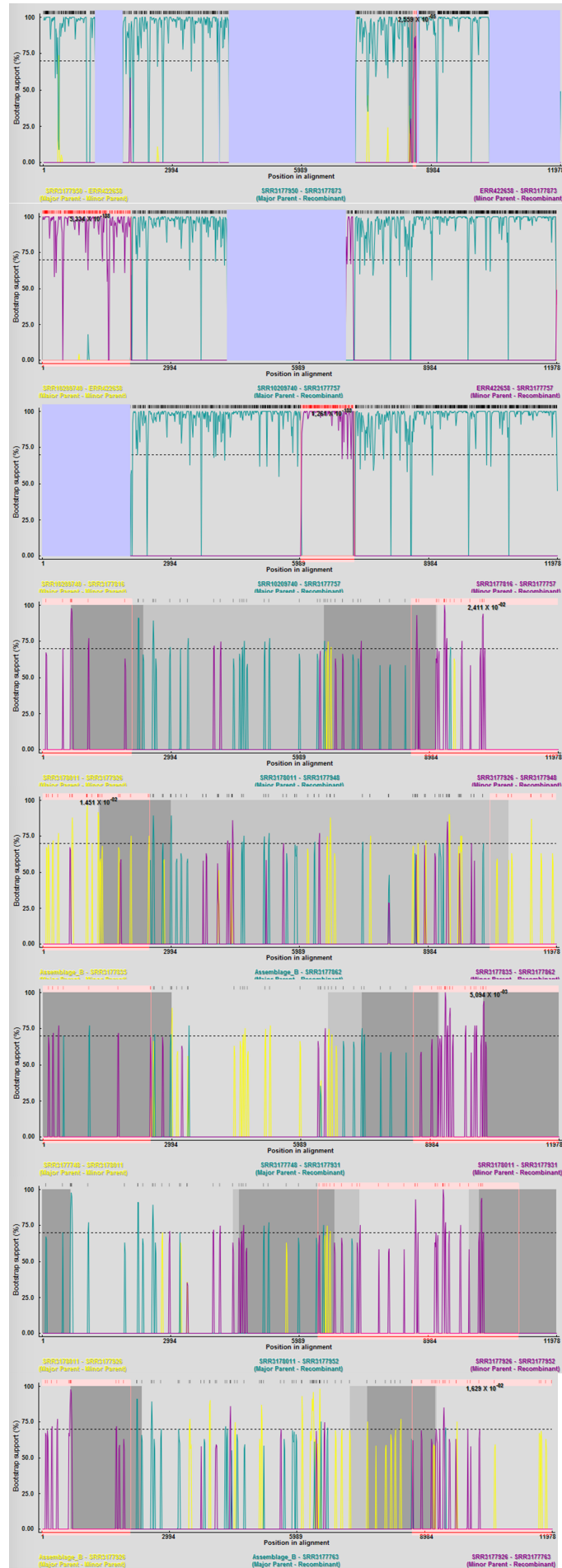

Supplement: Supplementary file 1 [file genes-11-00764-s001.zip › Figure S10.pdf]

A

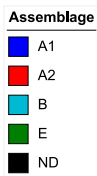

Tree scale: 0.01

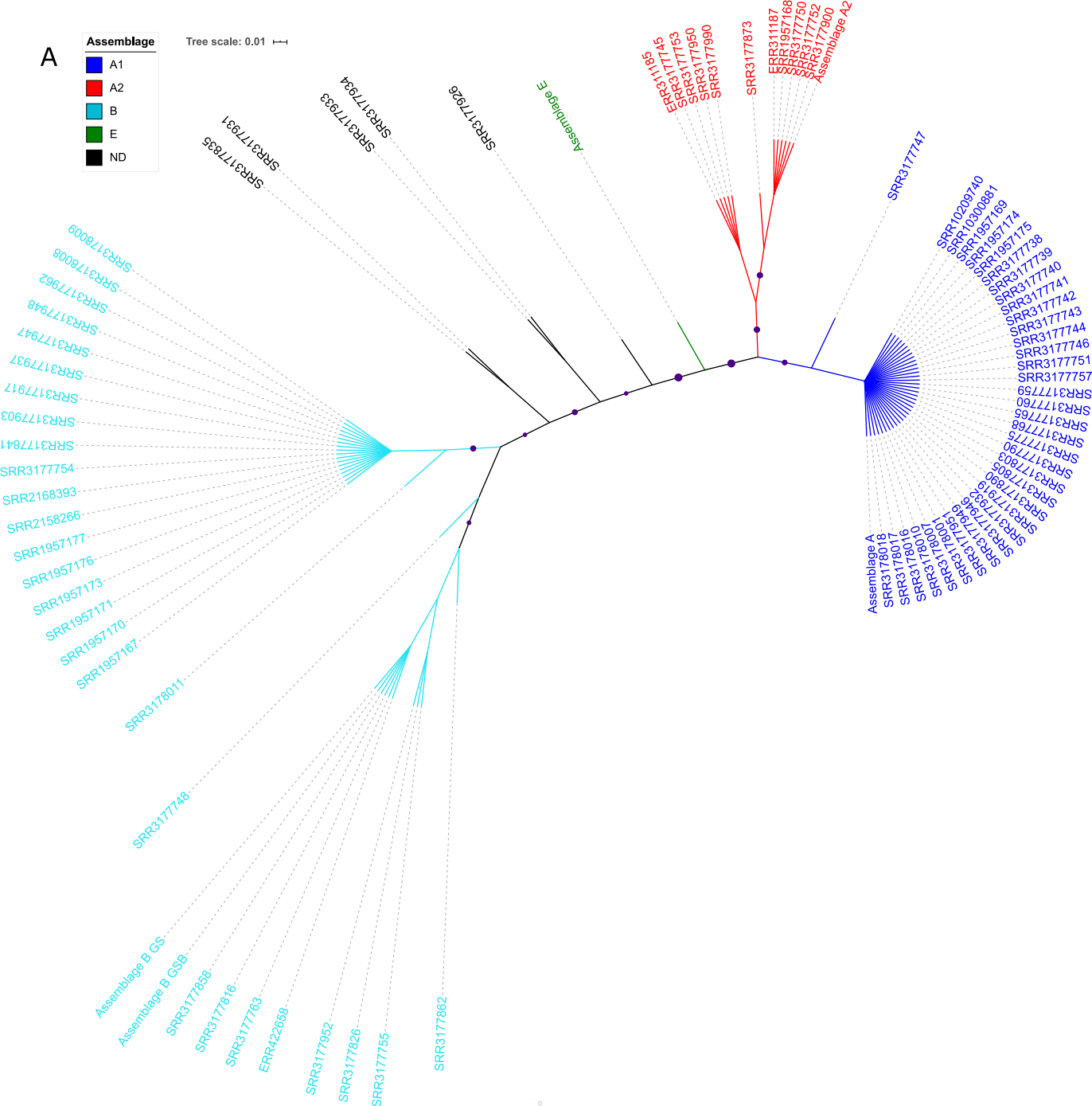

B

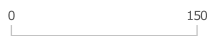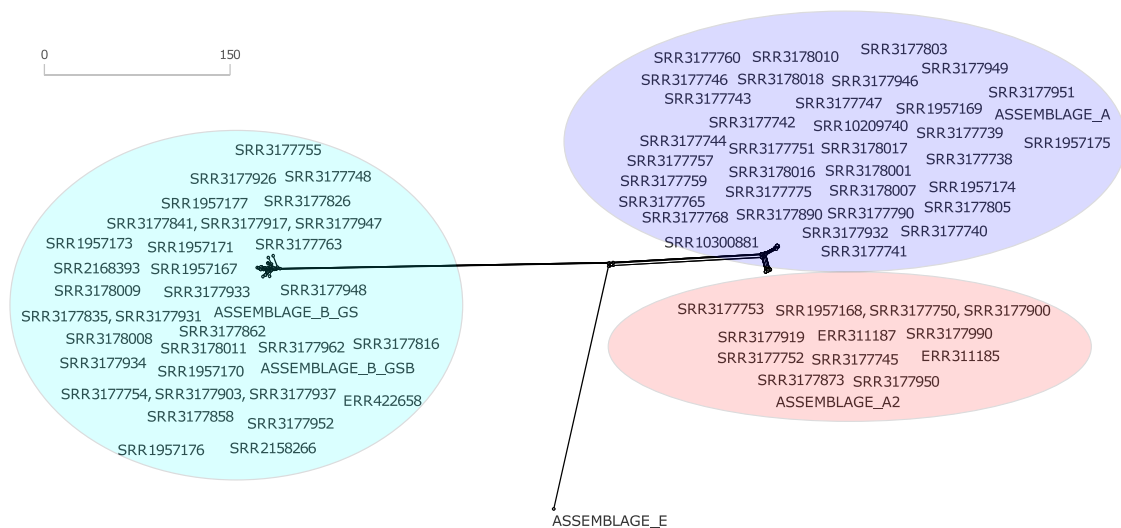

Supplement: Supplementary file 1 [file genes-11-00764-s001.zip › Figure S3.pdf]

I

## Assemblage

|                                                                                 |    |
|---------------------------------------------------------------------------------|----|
| 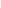   | A1 |
| 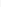   | A2 |
| 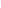  | B  |
| 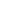 | E  |
| 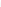 | ND |

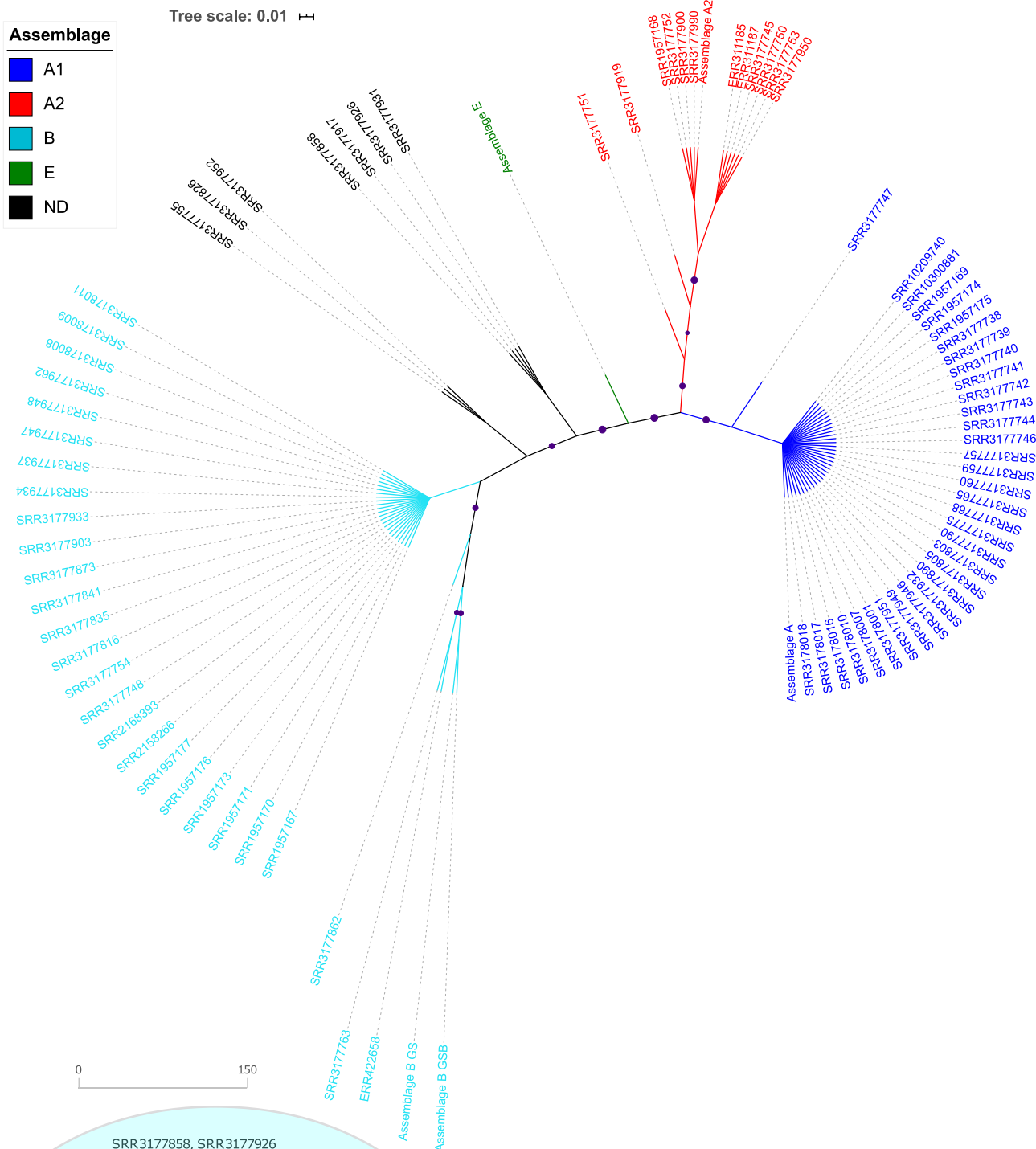

B

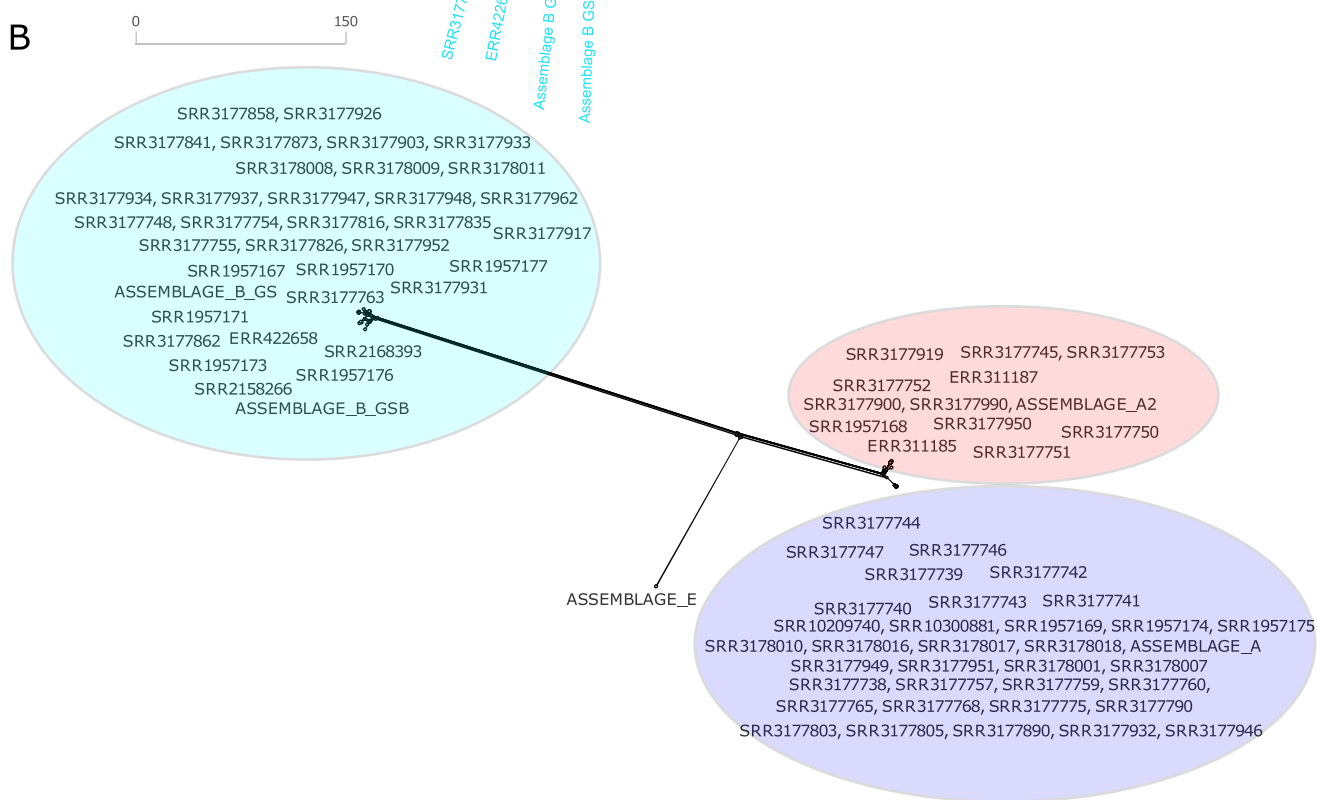

Supplement: Supplementary file 1 [file genes-11-00764-s001.zip › Figure S5.pdf]

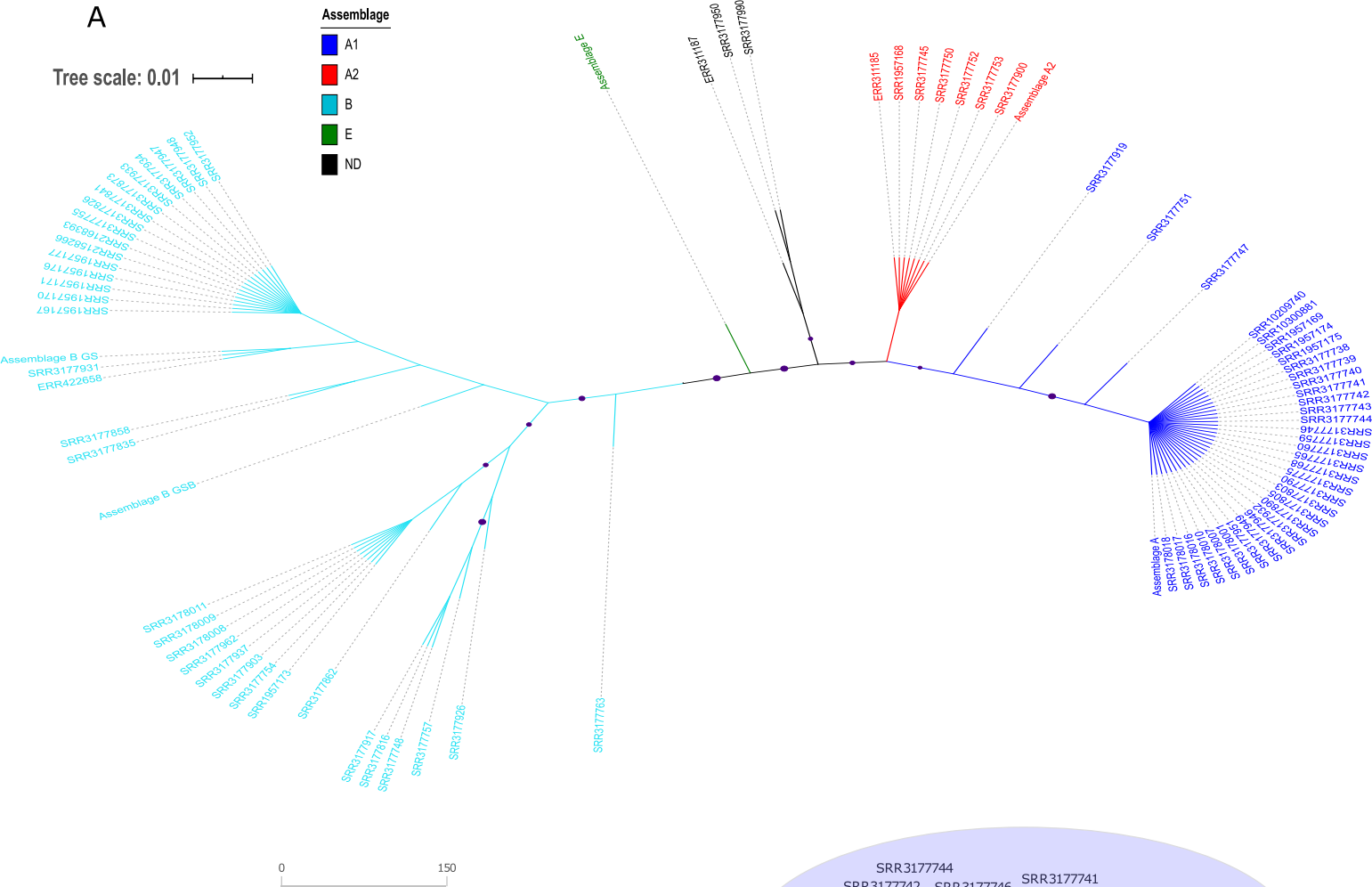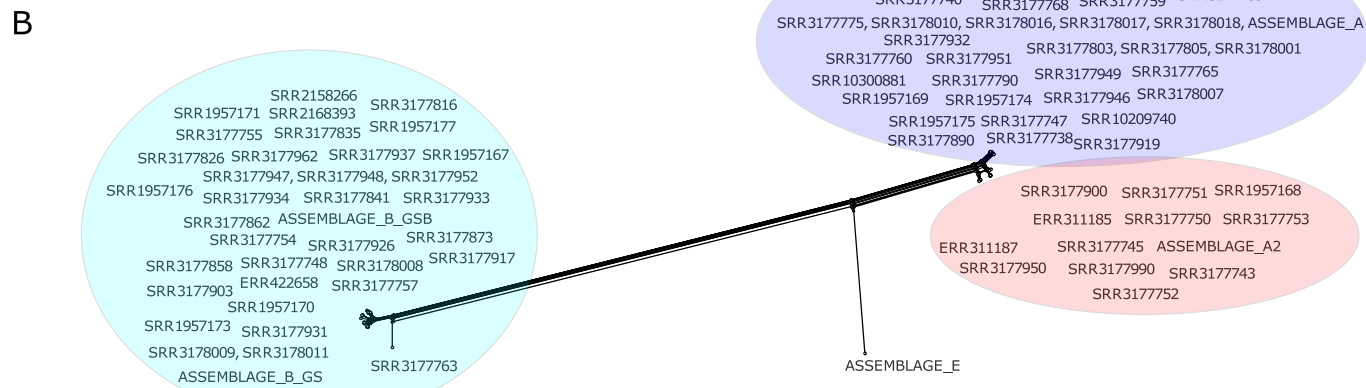

Supplement: Supplementary file 1 [file genes-11-00764-s001.zip › Figure S6.pdf]

A

Tree scale: 0.01

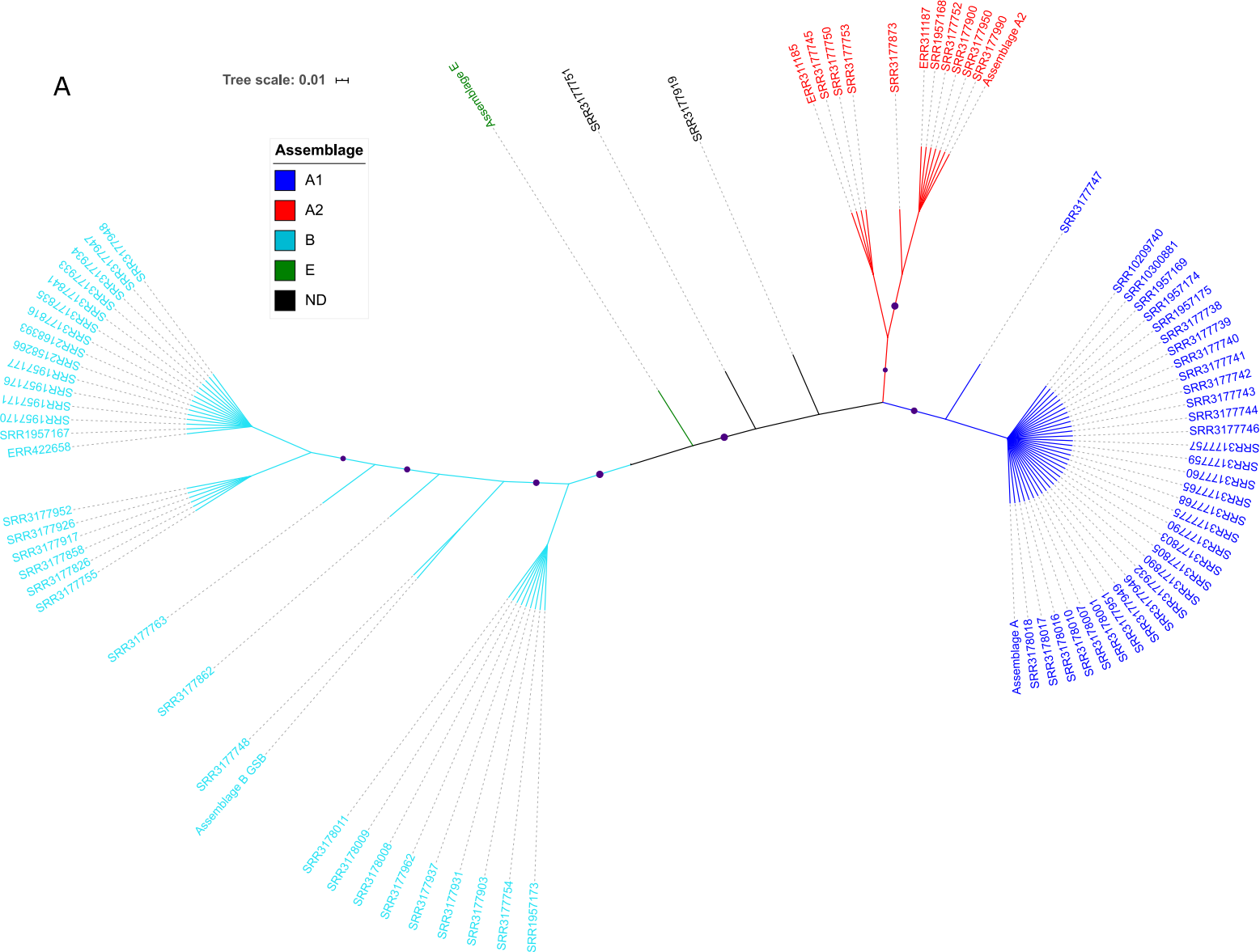

B

0 150

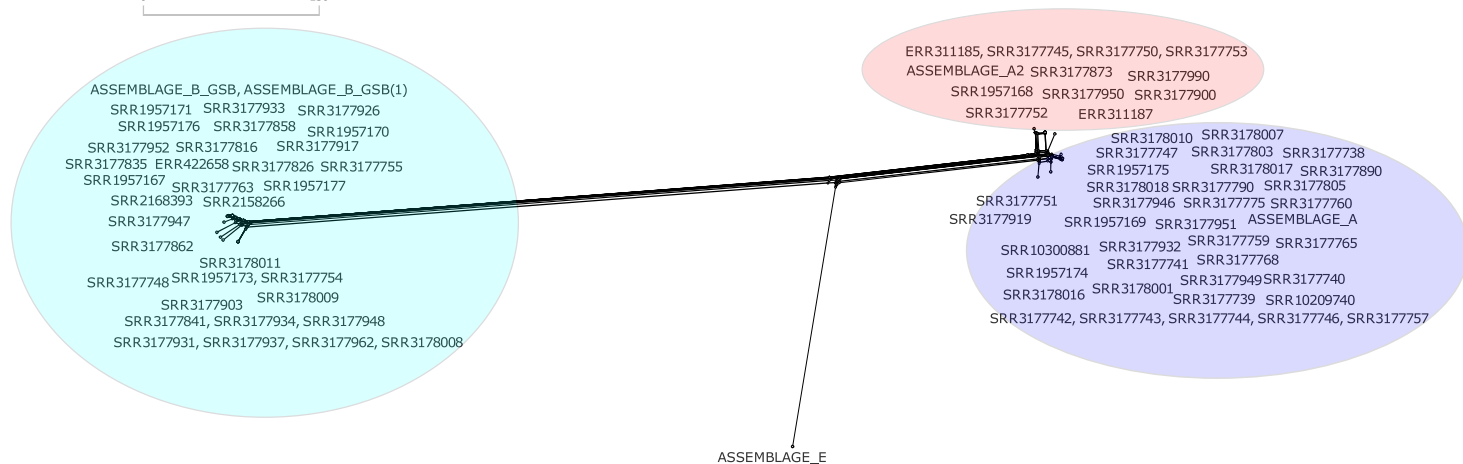

Supplement: Supplementary file 1 [file genes-11-00764-s001.zip › Figure S7.pdf]

A

Tree scale: 0.01

## Assemblage

- A1
- A2
- B
- E
- ND

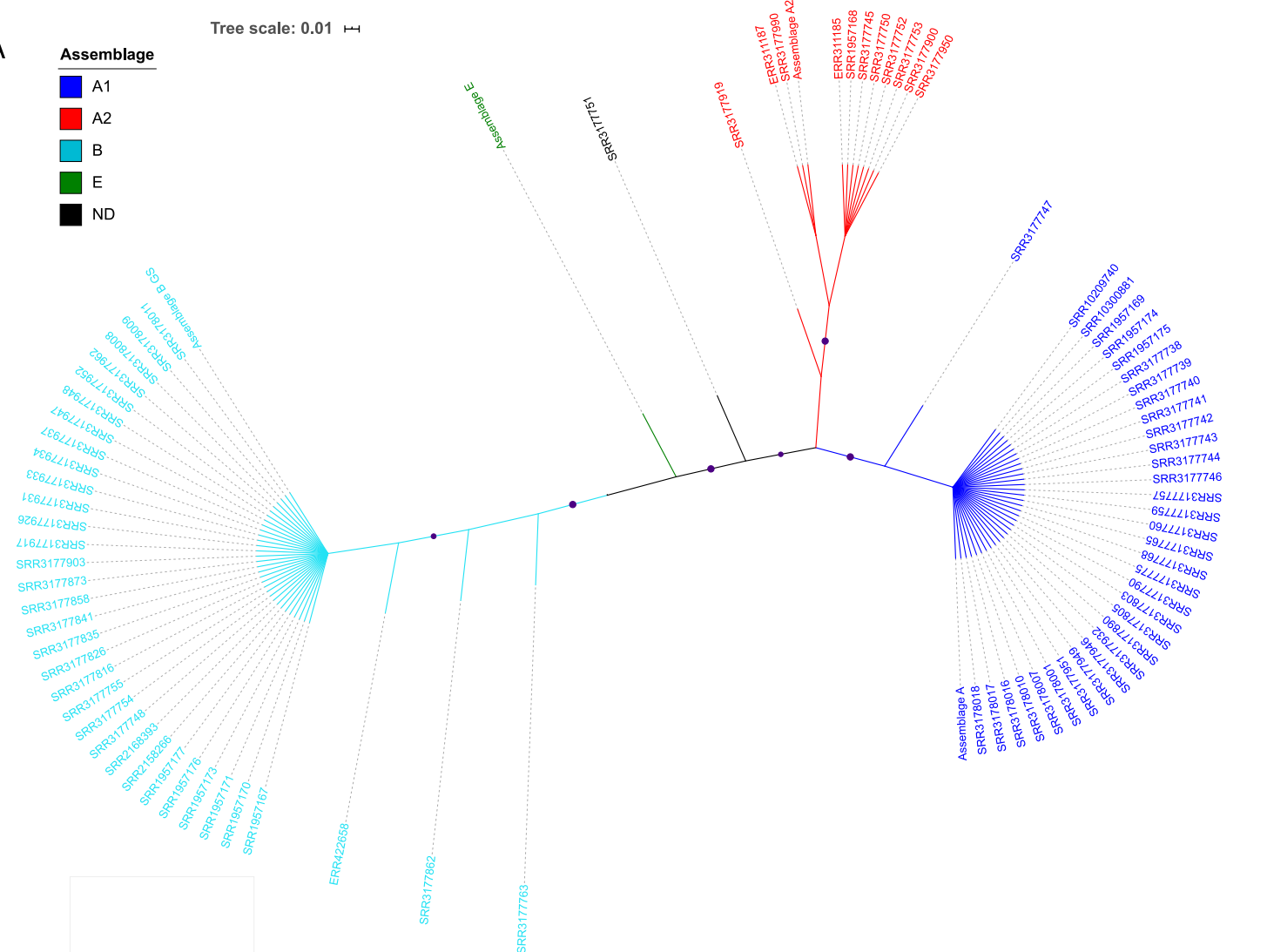

B

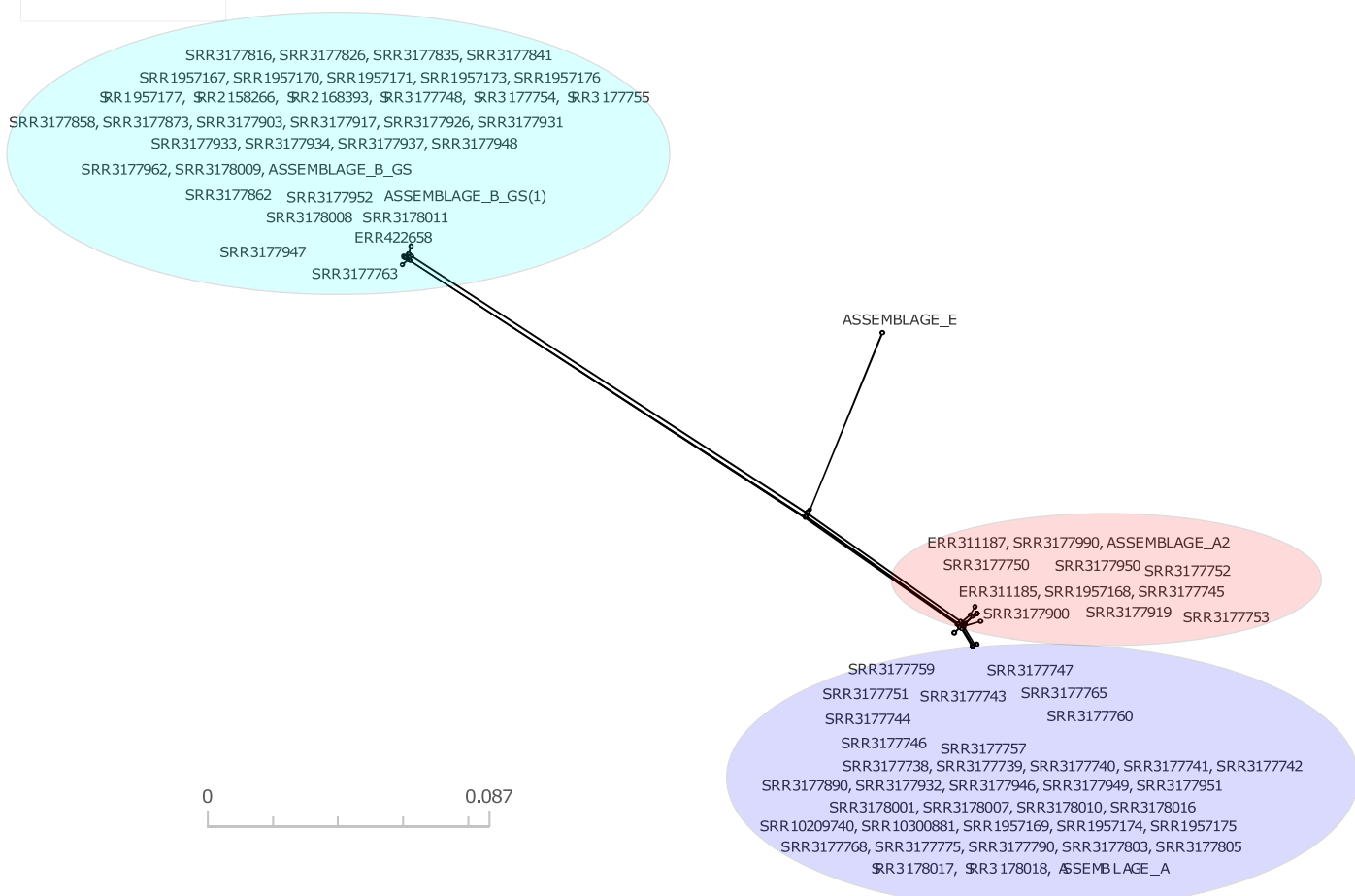

Supplement: Supplementary file 1 [file genes-11-00764-s001.zip › Figure S8.pdf]

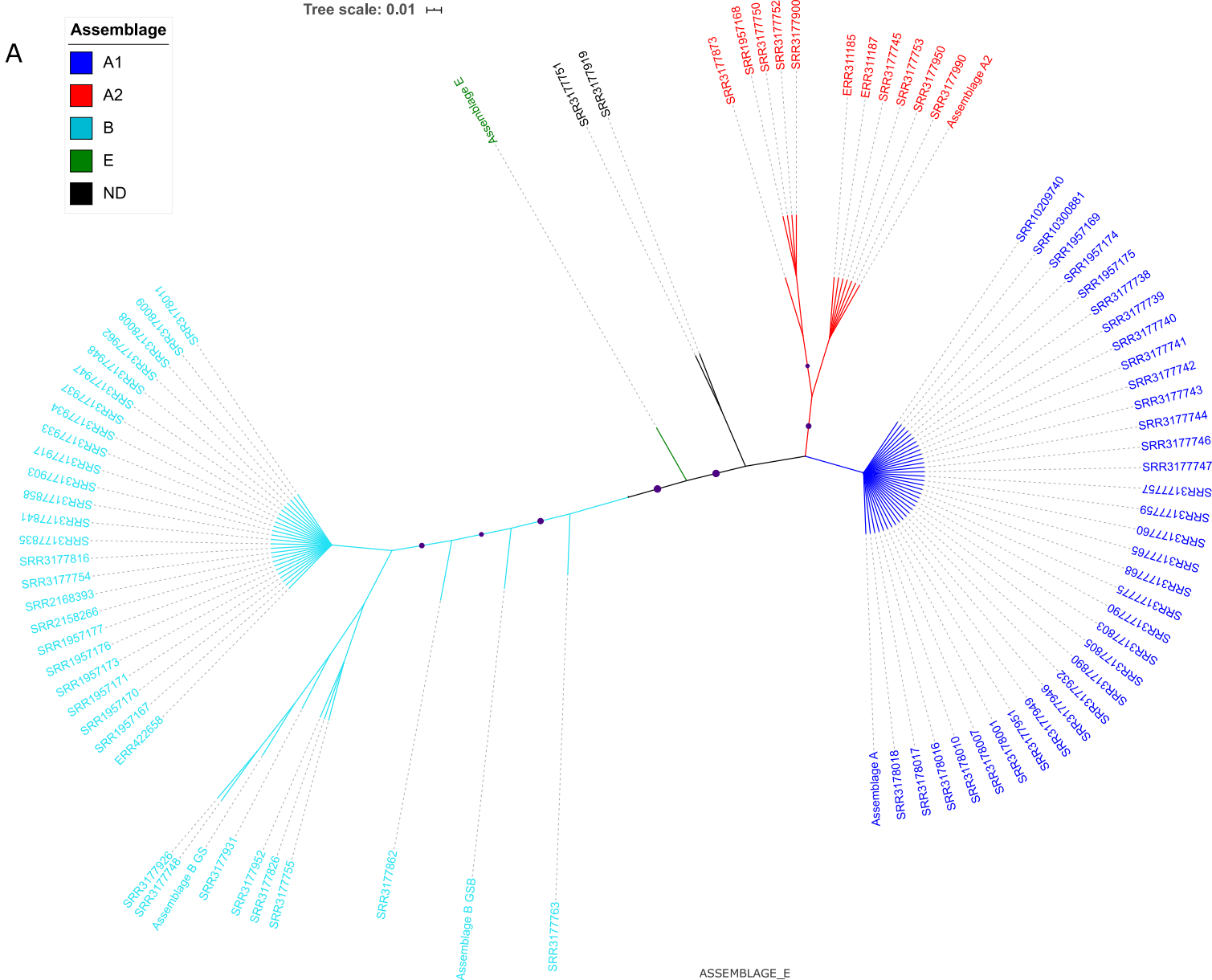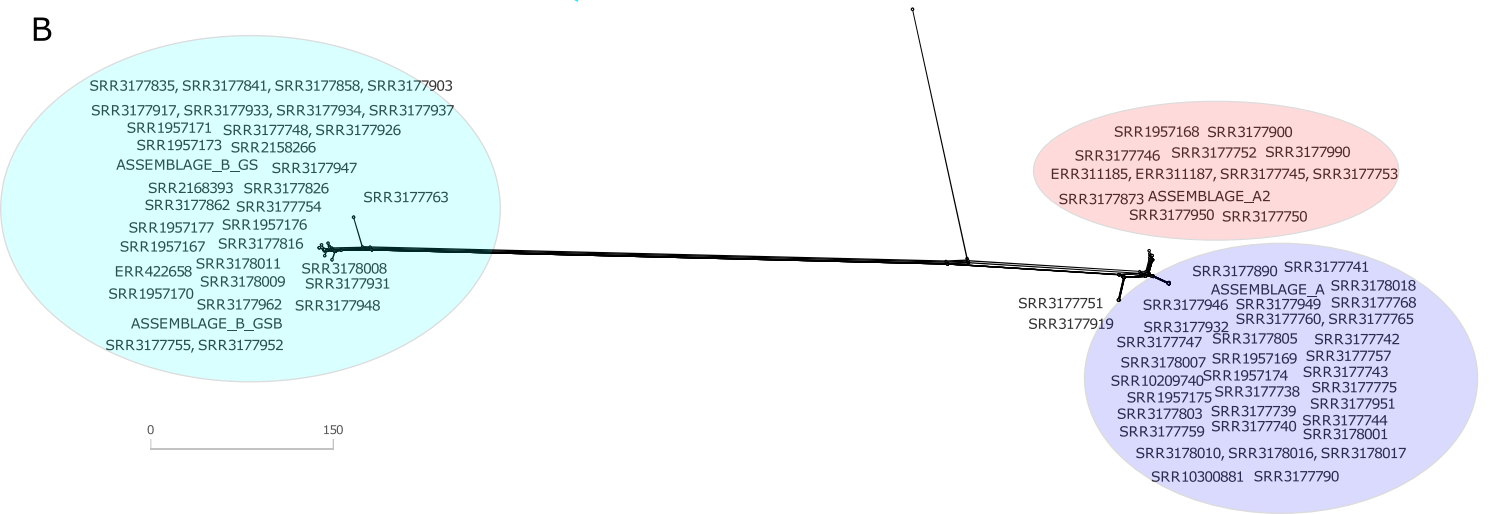

Supplement: Supplementary file 1 [file genes-11-00764-s001.zip › Figure S9.pdf]
