# Supplementary material for: Development of a Multilocus Sequence Typing Scheme for Giardia intestinalis
Source: Genes (Basel). 2020 Jul 8;11(7):764. doi: 10.3390/genes11070764 (PMC7397270; doi:10.3390/genes11070764)
Supplement: Supplementary file 1 [file Figure S4.pdf › Figure S4.pdf]

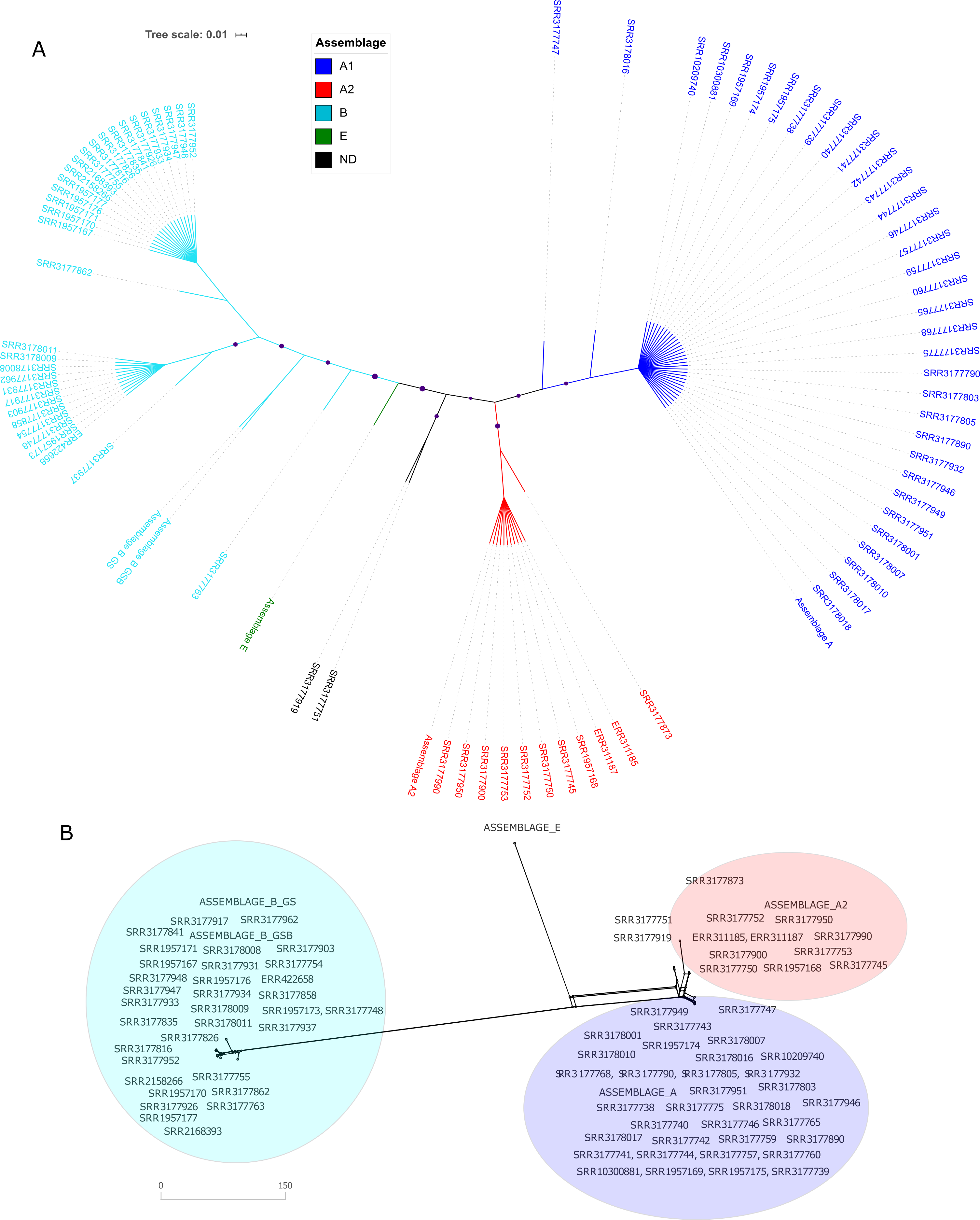

A
Tree scale: 0.01
Assemblage
A1
A2
B
E
ND
SRR3177747
SRR3178016
SRR10209740
SRR10300881
SRR1957169
SRR1957174
SRR1957175
SRR3177738
SRR3177739
SRR3177740
SRR3177741
SRR3177742
SRR3177743
SRR3177744
SRR3177746
SRR3177757
SRR3177759
SRR3177760
SRR3177765
SRR3177768
SRR3177775
SRR3177790
SRR3177803
SRR3177805
SRR3177890
SRR3177932
SRR3177946
SRR3177949
SRR3177951
SRR3178001
SRR3178007
SRR3178010
SRR3178017
SRR3178018
Assemblage A
SRR3177873
ERR311185
ERR311187
SRR1957168
SRR3177745
SRR3177750
SRR3177752
SRR3177753
SRR3177900
SRR3177950
SRR3177990
Assemblage A2
SRR3177751
SRR3177919
Assemblage E
SRR3177763
Assemblage B GSB
Assemblage B GS
SRR3177937
SRR3177862
SRR3178011
SRR3178009
SRR3178008
SRR3177962
SRR3177931
SRR3177917
SRR3177903
SRR3177858
SRR3177754
SRR3177748
SRR1957173
ERR422658
SRR1957167
SRR1957170
SRR1957171
SRR1957176
SRR1957177
SRR3177952
SRR3177948
SRR3177947
B
ASSEMBLAGE_E
ASSEMBLAGE_B_GS
SRR3177917 SRR3177962
SRR3177841 ASSEMBLAGE_B_GSB
SRR1957171 SRR3178008 SRR3177903
SRR1957167 SRR3177931 SRR3177754
SRR3177948 SRR1957176 ERR422658
SRR3177947 SRR3177934 SRR3177858
SRR3177933 SRR3178009 SRR1957173, SRR3177748
SRR3177835 SRR3178011 SRR3177937
SRR3177826
SRR3177816
SRR3177952
SRR2158266 SRR3177755
SRR1957170 SRR3177862
SRR3177926 SRR3177763
SRR1957177
SRR2168393
0
150
SRR3177873
ASSEMBLAGE_A2
SRR3177751 SRR3177752 SRR3177950
SRR3177919 ERR311185, ERR311187 SRR3177990
SRR3177900 SRR3177753
SRR3177750 SRR1957168 SRR3177745
SRR3177949 SRR3177747
SRR3177743
SRR3178001 SRR1957174 SRR3178007
SRR3178010 SRR3178016 SRR10209740
SRR3177768, SRR3177790, SRR3177805, SRR3177932
ASSEMBLAGE_A SRR3177951 SRR3177803
SRR3177738 SRR3177775 SRR3178018 SRR3177946
SRR3177740 SRR3177746 SRR3177765
SRR3178017 SRR3177742 SRR3177759 SRR3177890
SRR3177741, SRR3177744, SRR3177757, SRR3177760
SRR10300881, SRR1957169, SRR1957175, SRR3177739
